# Monocytic myeloid-derived suppressive cells mitigate over-adipogenesis of bone marrow microenvironment in aplastic anemia by inhibiting CD8^+^ T cells

**DOI:** 10.1038/s41419-022-05080-5

**Published:** 2022-07-18

**Authors:** Ying Qu, Zhengxu Sun, Yan Yuan, Zifeng Li, Fen Wang, Kunpeng Wu, Huihui Yu, Qiwang Lin, He Fei, Jian Chen, Maoxiang Qian, Yunfeng Cheng, Hua Jiang, Tong Chen

**Affiliations:** 1 Department of Hematology, Huashan Hospital, Fudan University, Shanghai, China; 2 Department of Hematology, The First Affiliated Hospital of Nanjing Medical University, Jiangsu Province Hospital, Nanjing, China; 3 Department of Hematology and Oncology, Children’s Hospital, National Children’s Medical Center, Fudan University, Shanghai, China; 4 Department of Gynecology, Obstetrics & Gynecology Hospital, Fudan University, Shanghai, China; 5 Department of Laboratory Medicine, Huashan Hospital, Fudan University, Shanghai, China; 6 Department of Hematology, Zhongshan Hospital, Fudan University, Shanghai, China

**Keywords:** Immunopathogenesis, Immunotherapy

## Abstract

Aplastic anemia (AA) is a blood disorder resulted from over-activated T-cell related hematopoietic failure, with the characterization of hypocellularity and enhanced adipogenic differentiation of mesenchymal stroma cells (MSCs) in bone marrow (BM). However, little is known about the relationship between immune imbalance and polarized adipogenic abnormity of BM microenvironment in this disease entity. In the present study, we differentiated BM-MSCs into osteoblastic or adipogenic lineages to mimic the osteo-adipogenic differentiation. Activated CD8^+^ T cells and interferon-γ (IFN-γ) were found to stimulate adipogenesis of BM-MSCs either in vitro or in vivo of AA mouse model. Interestingly, myeloid-derived suppressive cells (MDSCs), one of the immune-regulating populations, were decreased within BM of AA mice. We found that it was not CD11b^+^Ly6G^+^Ly6C^-^ granulocytic-MDSCs (gMDSCs) but CD11b^+^Ly6G^-^Ly6C^+^ monocytic-MDSCs (mMDSCs) inhibiting both T cell proliferation and IFN-γ production via inducible nitric oxide synthetase (iNOS) pathway. Single-cell RNA-sequencing (scRNA-seq) of AA- and mMDSCs-treated murine BM cells revealed that mMDSCs transfusion could reconstitute BM hematopoietic progenitors by inhibiting T cells population and signature cytokines and decreasing immature Adipo-Cxcl12-abundant reticular cells within BM. Multi-injection of mMDSCs into AA mice reduced intra-BM T cells infiltration and suppressed BM adipogenesis, which subsequently restored the intra-BM immune balance and eventually prevented pancytopenia and hypo-hematopoiesis. In conclusion, adoptive transfusion of mMDSCs might be a novel immune-regulating strategy to treat AA, accounting for not only restoring the intra-BM immune balance but also improving stroma’s multi-differentiating microenvironment.

## Introduction

Aplastic anemia (AA) is one type of bone marrow failure (BMF) characterized by pancytopenia in peripheral blood (PB) and hypocellular hematopoiesis in bone marrow (BM), which is resulted from over-activated T cells attack [1, 2]. The immune-suppressive medicines, antithymocyte globin (ATG) and cyclosporin A (CsA), have been successfully applied to treat AA patients. However, there are still 20–40% of the patients showing less response to immune-suppressive treatment (IST) [3, 4], indicating that it is necessary to deeply dissect the pathogenesis and to explore novel therapeutic strategies for this disease entity.

Functional hematopoiesis relies on not only abundant hematopoietic stem cells (HSCs) but also the balanced supportive hematopoietic niche. Intra-BM immune balance, at either cellular or cytokine level, is one of the key footstones to maintain a functional hematopoietic microenvironment. During AA progression, over-activated CD8^+^ T cells represent the immune-activating cellular part, while BM-mesenchymal stromal cells (MSCs), regulating T cells (Tregs) and myeloid-derived suppressive cells (MDSCs), sit on the immune-modulatory end. However, the current therapeutic point to treat AA, including IST^5^and HSCs transplantation [5, 6], focuses on rescuing HSCs from the attack of “BM-targeted” T cells. Little is known about the relationship between immune imbalance and abnormity of BM microenvironment [7, 8].

In fact, in addition to the absence of hematopoietic precursors in AA, the characterization of fat tissue in “empty” BM suggested that BM stroma is also affected during AA development. In AA mice models, administration of antagonist of peroxisomal proliferator-activated receptor gamma (PPARγ), a key transcription factor of adipogenesis, has been shown to reduce the infiltration of T cells into BM and thus to ameliorate BMF [9].

In this study, we focused on dissecting the abnormity of the intra-BM AA microenvironment in the presence or absence of suppressive MDSCs, whose function remains undefined in the AA process. We found that the multipotent BM-MSCs were polarized toward adipocytic differentiation under the stimulation of activated T cells and type 1 cytokine, interferon gamma (IFN-γ). The multi-injection of monocytic MDSCs (mMDSCs) into AA mice not only reduced intra-BM T cells infiltration but also suppressed BM adipogenesis, which subsequently restored the intra-BM immune balance and eventually prevented peripheral pancytopenia and hypo-cellularity within BM.

## Results

### Over-adipogenic differentiation of BM-MSCs and decreased MDSCs were detected in AA mice

**Fig. 1:**
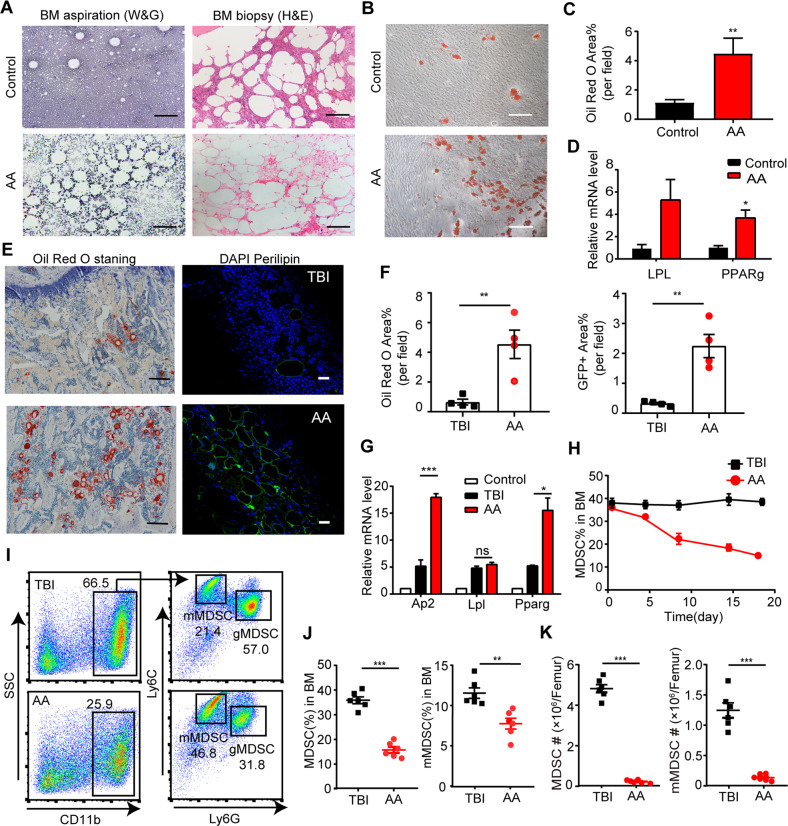
The polarized adipogenic differentiation of BM-MSCs and decreased MDSC populations in AA mice. **A** Representative Wright-Giemsa staining of BM aspiration (left panel) and H&E staining of BM biopsy (right panel) from AA patients and controls. Scale bar:100 μm. **B** Images of representative Oil Red O staining of adipogenic induced BM-MSCs from AA patients and controls. Scale bar:200 μm. **C** Percentage of the area showing Oil Red O staining cells per field in the BM image of AA patients and controls on day14 of MSCs’ adipogenic differentiation. Data were presented as the mean ± SD. *N* = 6 in each group. Independent experiments were repeated three times. ***P < 0.01. **D** Quantitative RT-PCR analysis of adipogenic markers (*LPL, PPARg*) in BM-MSCs of AA patients and controls. Data represent mean ± SEM in three independent experiments. *N* = 6 in each group. *P < 0.05. **E** Oil Red O staining and fluorescent staining of perilipin (green) and DAPI (blue) were performed on femur bone section from AA mice and TBI mice. Scale bar:50 μm. **F** Percentage of the area showing Oil Red O staining cells (left) and GFP^+^ Perilipin staining ^(^right) per field in the BM image of TBI mice and AA mice on day14 of MSCs’ adipogenic differentiation. Data were presented as the mean ± SD. *N* = 4 in each group. **P < 0.01. **G** Relative expression of *Ap2, Lpl*, and *Pparg* in BM from AA and TBI mice were determined. Data represent mean ± SEM in three independent experiments. *N* = 6 in each group. *P < 0.05, ***P < 0.001. **H** The percentage of MDSCs in BM of TBI mice and AA mice were detected after irradiation and LN cell injection. Data were presented as the mean ± SD. *N* = 3 in each group at each time point. **I** Representative immunophenotypic analysis of BM MDSCs, mMDSCs, and gMDSCs in TBI and AA mice on day 14. The percentage (**J**) and ANs (**K**) of MDSCs, and mMDSCs in BM were determined from AA and TBI mice. Data were presented as the mean ± SD. *N* = 6 in each group. **P < 0.01,***P < 0.001.

BM histological examinations in AA patients showed significant hypoplasia with the fat tissue replacement in the marrow space (Fig. 1A). As expected, BM-MSCs from AA patients exhibited increasing Oil Red O staining and expression of adipocyte-related genes, *Lpl* and *Ppar*, indicating an enhanced adipogenic differentiating capability (Fig. 1B–D). Similar features were observed in AA mouse models. The intra-BM staining of Oil Red O and perilipin were dramatically increased in murine AA-BMs (Fig. 1E, F), where the expression of *Ap2*, *Lpl*, and *Pparγ* were upregulated coincidentally (Fig. 1G).

Considering the negative immune regulating function of MDSCs, we investigated the proportion of MDSCs populations within murine AA-BM. Compared with TBI mice, the whole population of intra-BM MDSCs (CD11b^+^Gr-1^+^) decreased along with the disease progression (Fig. 1H). Either the percentages or the absolute numbers (ANs) of CD11b^+^Gr-1^+^ MDSCs (percentage: 16.5 ± 1.258 *vs* 37.17 ± 1.447, *P* < 0.001; AN: 4.861 ± 0.190/femur *vs* 0.273 ± 0.031/femur, *P* < 0.001) and CD11b^+^Ly6G^−^Ly6C^+^ mMDSCs (percentage: 7.745 ± 0.643 vs 11.41 ± 0.621, *P* < 0.01; AN: 1.246 ± 0.123/femur vs 0.138 ± 0.022/femur, *P* < 0.001) in AA mice’ BM were significantly decreased compared to the TBI mice (Fig. 1I–K), suggesting that MDSC and mMDSC populations were diminished in AA process.

### CD8^+^ T cells were the major population infiltrated into recipients’ BM and induced BMF in AA mice

**Fig. 2:**
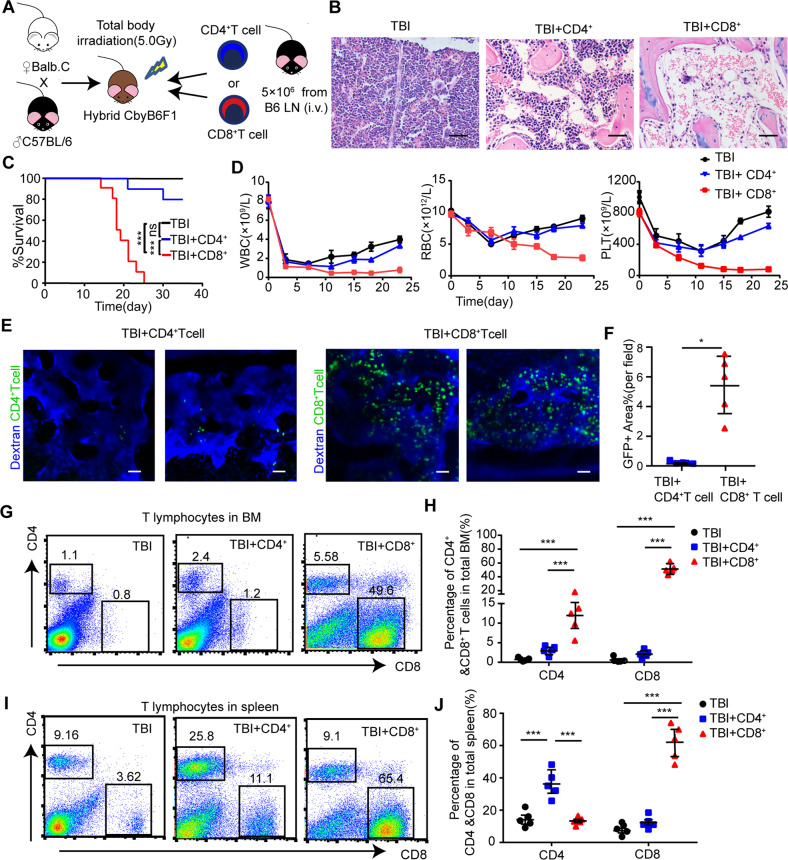
BMF features of AA mouse model were majorly induced by CD8^+^ T cells. **A** Experimental scheme of generating AA mice. TBI irradiated (5.0 Gy) CbyB6F1 mice were injected with 5 × 10^6^ CD4^+^ or CD8^+^ T cells from B6 mice lymph node via tail vein injection. **B** Images of representative H&E staining of murine femurs in different groups were taken under microscope. Scale bar:100 μm. **C** The survival curves were determined in pre-irradiated (5.0 Gy) CbyB6F1 mice (TBI), TBI mice injected with 5 × 10^6^ CD4^+^ T cells (TBI + CD4^+^) or CD8^+^ T cells (TBI + CD8^+^). *N* = 10 in each group. Data were pooled from three independent experiments. ***P < 0.001. **D** Blood counts of TBI mice, TBI mice injected with CD4^+^T cells or CD8^+^ T cells. Data were presented as the mean ± SD. *N* = 5 in each group at each time point. **E** Representative in vivo image of skull BM in living mice injected with CD4^+^ T cells or CD8^+^ T cells. Pre-irradiated CbyB6F1 mice received 5 × 10^6^ GFP^+^CD4^+^ or GFP^+^CD8^+^ T cells. Vessel was stained with dextran-CY5 (blue) by tail vein injection. GFP^+^ infiltrating lymphocytes (green) were visualized under microscope. Scale bar:100 μm. **F** Percentage of the area showing GFP^+^ T cells per field in the image of intravital skull BM cavity from the mice injected with CD4^+^ T cells or CD8^+^ T cells on day14. Data were presented as the mean ± SD. *N* = 5 in each group. *P < 0.05. FACs analyses of representative dot plot (**G**) and mean±SD (**H**) of CD4^+^ T cells and CD8^+^ T cells in BM of TBI mice, TBI mice injected with CD4^+^T cells, and TBI mice injected with CD8^+^ T cells on day14. FACs analyses of representative dot plot (**I**) and mean±SD (**J**) of CD4^+^ T cells and CD8^+^ T cells in the spleen of TBI mice, TBI mice injected with CD4^+^T cells and TBI mice injected with CD8^+^ T cells on day14. *N* = 5 in each group. ***P < 0.001.

In order to distinguish which T cell population causes immune-mediated AA, we injected 5 × 10^6^ B6-derived CD4^+^ or CD8^+^ T cells in pre-irradiated CByB6F1 mice (Fig. 2A). Pathological analysis clarified that BMs of CD8^+^ T cell-injected AA mice exhibited severe hypocellularity and adipocytic accumulation, while CD4^+^ T cell-treated mice did not (Fig. 2B). Fifty percent overall survival (OS) of CD8^+^ T cell-injected mice was at 18.5 days, while almost all the mice in CD4^+^ T cell-injected group and TBI group survived during the entire 35 days of follow-up (Fig. 2C). The blood cells were kept at the lowest level in CD8^+^ T cell-injected mice while CD4^+^ T cell-injected and TBI mice recovered (Fig. 2D).

Ten days after tail vein injection, GFP^+^ donor-derived CD8^+^ T cells were significantly infiltrated into recipients’ skull BM compared to CD4^+^ T cells (Fig. 2E, F). Relative to controlled mice receiving TBI and CD4^+^ T cells, CD8^+^ T cells-injected mice showed significant expansion of CD8^+^ T cells either in the BM (Fig. 2G, H) or in the spleen (Fig. 2I, J). A dramatically inverted ratio of CD4^+^/CD8^+^ T cells of BM and spleen was seen in CD8^+^ T cell-injected mice rather than in TBI mice and TBI with CD4^+^ T cell-injected mice at 1.5–2.5:1 (Fig. 2G–J). All these data indicated that CD8^+^ T cells exert a key function in inducing AA progression.

### IFN-γ enhanced adipogenic differentiation of BM-MSCs

**Fig. 3:**
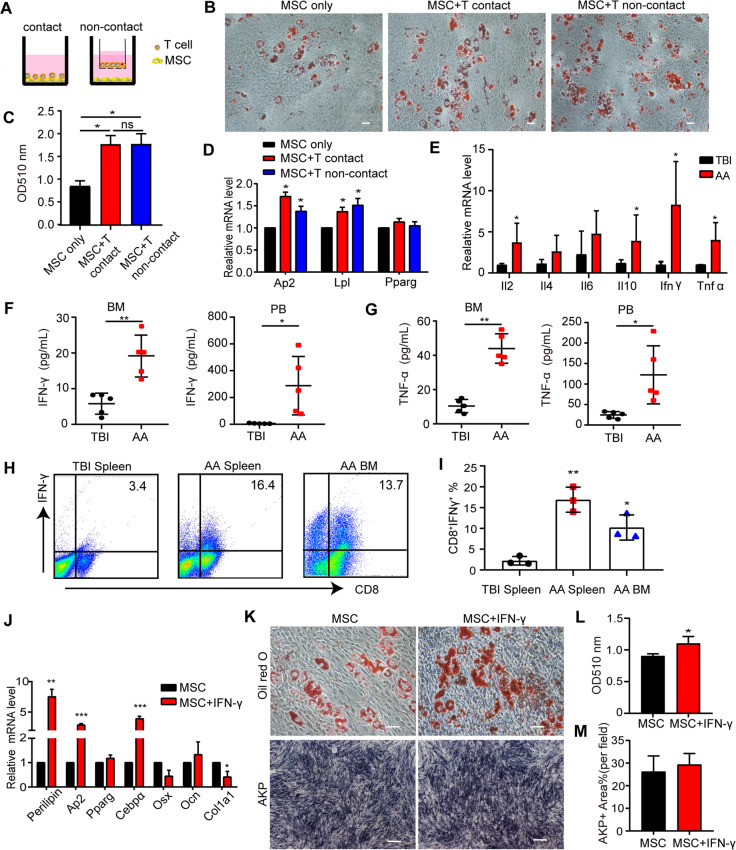
BM-MSCs were differentiated into adipogenic lineage by ConA-activated T cells and IFN-γ. **A** Outline of coculture system relating to direct contact model of BM-MSCs with CD3^+^ T cells (left), or non-contact model (right) in 0.4 μm pore size of transwell system, where murine CD3^+^ T cells were plated in the upper chamber and BM-MSCs were in the lower chamber. Oil Red O staining (**B**) and quantitative OD value at 510 nm (**C**, mean±SEM) in the presence or absence of CD3^+^ T cells after 14 days of adipogenic differentiation of murine BM-MSCs. Scale bar:50 μm. *P < 0.05. **D** Expression of adipogenic markers (*Ap2, Lpl,* and *Pparg*) of murine BM-MSCs in the models of direct-contact or non-contact were analyzed 14 days after adipogenic induction. Data represented mean ± SEM in three independent experiments. *P < 0.05. **E** Expression of IL-2, IL-4, IL-6, IL-10, IFN-γ, and TNF-α in BM cells of TBI and AA mice were determined by qRT-PCR. Data represent mean ± SEM in three independent experiments. *P < 0.05. The concentrations of IFN-γ (**F**) and TNF-α (**G**) in the supernatant of 500 μL/femur BM flushing fluid and in the serum of TBI mice and AA mice on day 14 were assessed by CBA kit. Data were presented as the mean ± SD. *N* = 5 in each group. *P < 0.05, **P < 0.01. FACs analyses of representative dot plot (**H**) and mean±SD (**I**) of intracellular staining of IFN-γ in murine CD8^+^ T cells from TBI spleen, AA spleen, and AA BM. Data were representative of three independent experiments. **J** Expression of adipocytic markers (*Perilipin, Lpl*, *Cebpα*, *Pparg*) and osteoblastic markers (*Osx*, *Ocn*, and *Col1a1*) in the presence or absence of IFN-γ of murine BM-MSCs was assessed by qRT-PCR after adipogenic/osteogenic induction. Data represent mean±SEM in three independent experiments. *P < 0.05, **P < 0.01, ***P < 0.001. **K**–**M** BM-MSCs were incubated with IFN-γ (10 ng/mL) for 72 h, and then cultured in differentiation media. **K** Staining of Oil Red O or AKP was performed on BM-MSCs 14 days after adipogenic or osteogenic differentiation respectively, in the presence or absence of IFN-γ. Scale bar: 50 μm. OD value at 510 nm of Oil Red O staining (**L**) and area of AKP-positive staining (**M**) were determined. Data represent mean±SEM in three independent experiments. *P < 0.05.

We then investigated the relationship between pathogenic intra-BM T cell infiltration and polarized adipocytic differentiation of BM-MSCs in AA mice. In a manner of either direct contact or indirect contact of transwell culture system (Fig. 3A), CD3^+^ T cells were confirmed to promote adipocytic differentiation of MSCs by Oil Red O staining (Fig. 3B, C) and adipocytic gene expression (Fig. 3D), suggesting that the adipogenic fate-shift of BM-MSCs is caused by activated T cells and T cell-secreting cytokines.

To dissect the relationship between the T cell-secreting cytokines and AA-related adipocytic BM-MSC differentiation, we analyzed the Th1/2 cytokine profile in PB and BM from AA mice and TBI controls. Expression of IFN-γ, TNF-α, IL-2, IL-4, and IL-10 were increased in AA-BM cells (Fig. 3E). Consistent with previous observation [10, 11], the concentrations of IFN-γ (19.20 ± 2.66 pg/mL vs 5.79 ± 1.32 pg/mL, Fig. 3F) and TNF-α (43.96 ± 3.85 pg/mL *vs* 10.41 ± 1.71 pg/mL, Fig. 3G) were significantly elevated at day 14 in the total 500μl of AA-BM flushing fluid compared with TBI-BM. Interestingly, both IFN-γ and TNF-α were higher in AA-PB than in AA-BM (Fig. 3F, G). No differences of IL-2, IL-4, and IL-10 concentration were seen between AA and TBI mice except for higher IL-6 in AA-PB (Supplementary Fig. 1A–D). Consistently, IFN-γ-expressing CD8^+^ T cells were increased in the AA-spleen and AA-BM (Fig. 3H, I).

To further determine the function of elevated IFN-γ and TNF-α on BM adipogenesis, BM-MSCs were incubated with IFN-γ or TNF-α for 72 h before adipogenic induction. IFN-γ-pretreated BM-MSCs showed increased expression of adipogenic genes (*Ap2, Perilipin,* and *Cebpα*) and Oil Red O staining (Fig. 3J–L). However, there was no difference of osteogenic genes (*Osx, Ocn*, and *Col1a1*) expression and AKP staining in the presence of IFN-γ (Fig. 3J, K, M). Interestingly, TNF-α interfered both adipogenic and osteoblastic differentiation of BM-MSCs (Supplementary Fig. 1E–H). All these results indicated that IFN-γ accelerated the adipocytic fate-shift during MSCs differentiation at the expense of reducing the osteogenic potential.

### Monocytic MDSCs inhibited T cell proliferation *via* iNOS pathway in vitro

**Fig. 4:**
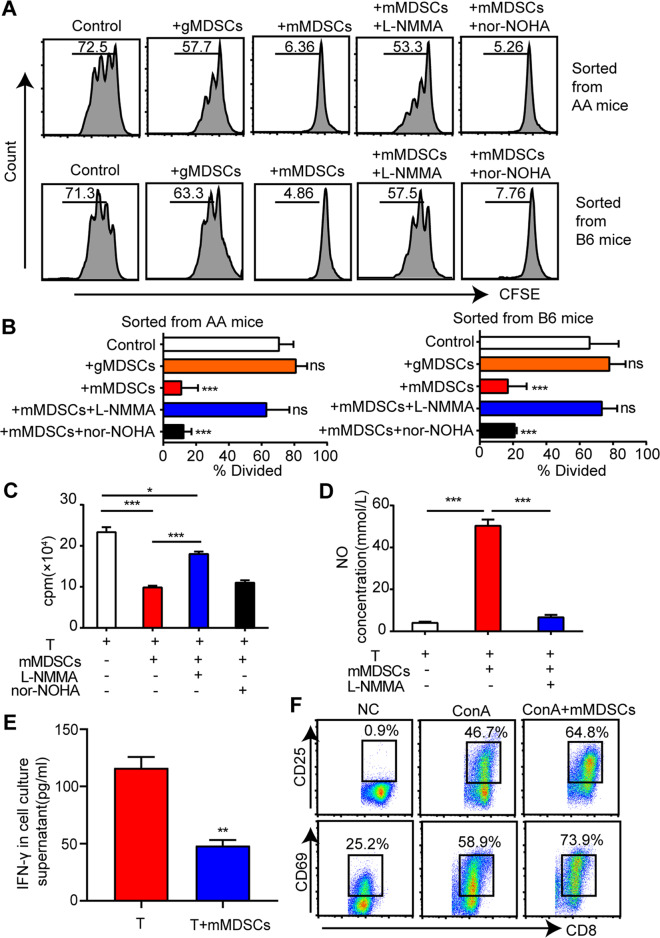
The effect of mMDSCs on T cell proliferation and IFN-γ secretion in vitro. **A**, **B** CFSE-labeled CD8^+^ T cells were activated with ConA (5 μg/mL) for 72 h, and cocultured with AA-BM derived mMDSC or gMDSC at the ratio of 1:1. Induced NOS inhibitor L-NMMA (2 μg/mL) and Arg-1 inhibitor nor-NOHA (2 μg/mL) were added in the culture system. **A** Data were collected from BM cells of AA mice or healthy B6 mice, with representative FACs analyses. **B** Histograms represented the percentages of CD8^+^ T cells that divided into different treatment groups. Data represent mean±SEM in three independent experiments. ***P < 0.001. **C** The numbers of CD8^+^ T cells in co-culture system were counted by flow cytometer at the same in-flux rate per minute, with or without mMDSCs, or with mMDSCs and L-NMMA/nor-NOHA. Data represent mean ± SEM in three independent experiments. *P < 0.05, ***P < 0.001. **D** The NO concentration in the supernatants of ConA-stimulated T cells cocultured with mMDSCs (1:1), in the presence or absence of L-NMMA (2 μg/mL). Data represent mean ± SEM in three independent experiments. ***P < 0.001. **E** ConA-stimulated CD8^+^ T cells were cultured alone or co-cultured with mMDSCs at the ratio of 1:1 for 72 h. The concentrations of IFN-γ in the supernatants were determined. Data represent mean±SEM in three independent experiments. **P < 0.01. **F** FACs analysis of T cell activated makers, CD25 and CD69 on ConA-stimulated CD8^+^ T cells cocultured with or without AA-BM derived mMDSCs for 72 h.

By the analysis of carboxyfluorescein diacetate succinimidyl ester (CFSE)-labeled cells, mMDSCs were found to inhibit CD8^+^ T cell proliferation in an ex vivo culture system (Supplementary Fig. 2A, B). To identify whether mMDSCs exert inhibition owing to expression of arginase-1(Arg-1) or induced nitric oxide synthase (iNOS) [12], the respective antagonist of iNOS and Arg-1 pathway, L-NMMA, and Nor-NOHA, were added to the culture system. The addition of L-NMMA in the mMDSCs system restored the T cell proliferation whereas Nor-NOHA did not, demonstrating that the inhibiting function is via the iNOS pathway (Fig. 4A, B). The concentration of NO in the supernatant of T cells co-culturing with mMDSCs was increased while L-NMMA significantly reduced mMDSCs-enhanced NO production (Fig. 4C, D). These data confirmed that the iNOS pathway plays a significant role in the immune-modulate properties of mMDSCs. In the co-culture system of mMDSCs with ConA-stimulated T cells, IFN-γ in the supernatant was almost half-reduced from 116.20 ± 9.44 to 50.40 ± 5.06 pg/mL (Fig. 4E). However, expression of the T cell activating markers, CD69 and CD25 showed no difference on ConA-activated T cells with or without mMDSCs, indicating that mMDSCs exert no effect on T cell activation (Fig. 4F).

### Monocytic MDSCs ameliorated pancytopenia and intra-BM lymphocytic infiltration in AA mice

We further determined whether adoptive transfusion of mMDSCs reconstitute the impacted hematopoiesis in the AA process. A single injection of 5 × 10^6^ mMDSCs at day 0 could not restore peripheral blood counts and did not improve survival rate or prevent hypo-cellularity within BM (Supplementary Fig. 3A–C). A temporary recovery from BM hypo-cellularity and decreased intra-BM CD8^+^ T cell infiltration were found on day 7 in AA mice injected with a single dose of mMDSCs (Supplementary Fig. 3C–E). But the infiltrated CD8^+^ T cells within BM were re-elevated to the untreated level on day 14 (Supplementary Fig. 3D, F). And the intra-BM ratio of CD4^+^/CD8^+^ T cells in mMDSCs-treated mice was reversed on day 14 (Supplementary Fig. 3E, F), suggesting that single-dose of mMDSCs might only be able to prevent CTL infiltration and proliferation in a very short term.

**Fig. 5:**
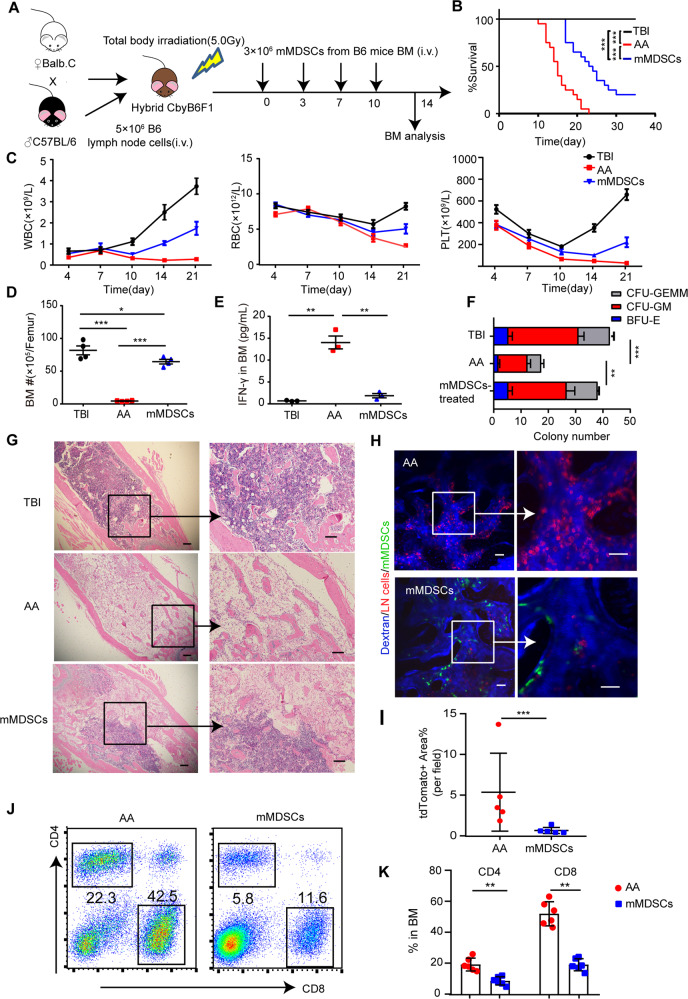
Monocytic MDSCs reduced lymphocytic infiltration and IFN-γ secretion in murine AA-BM. **A** Schematic outline of adoptive mMDSCs treatment in AA mice. Pre-irradiated CbyB6F1 mice were injected with 5 × 10^6^ B6-LN cells to induce AA, and treated with 3 × 10^6^ mMDSCs on day 0, 3, 7, and 10. **B** The survival curves were determined in pre-irradiated (5.0 Gy) CbyB6F1 mice (TBI), TBI mice injected with 5 × 10^6^ LN cells (AA), AA mice injected with 3 × 10^6^ mMDSCs on day 0, 3, 7, 10 (mMDSCs treated). *N* = 20 in each group. Data were pooled from three independent experiment. ***P < 0.001. **C** The blood counts were determined in mice of TBI, AA, and mMDSCs-treated groups at different time points. Data were presented as the mean ± SD. *N* = 5 in each group. Total nucleated cell numbers per femur (**D**, *n* = 5) and IFN-γ concentration in 500 μL BM flushing fluid/femur (**E**, *n* = 5) were determined on day 14. Data were presented as the mean ± SD. **F** BM hematopoietic colony-forming unit (CFU) from TBI mice, AA mice, and mMDSC-treated mice in Methocult assays (BFU-E: burst-forming unit-erythroid; CFU-GM: CFU-granulocyte macrophage; CFU-GEMM: CFU-granulocyte, erythrocyte, macrophage, megakaryocyte). Data were presented as the mean ± SD. *N* = 4 in each group. **P < 0.01, ***P < 0.001. **G** H&E staining of femur BM from TBI mice, AA mice and mMDSC-treated AA mice on day 14. Scale bar: 50 μm. **H** In vivo imaging of recipients’ skull BM. Pre-irradiated CbyB6F1 mice received 5 × 10^6^ LN cells derived from tdTomato^+^ mT/mG mice (red), and treated with 3 × 10^6^ GFP-expressing mMDSCs on day 0, 3, 7, and 10. Vessels were stained by tail vein injection of dextran-CY5 (blue). TdTomato^+^ LN cells (red) and GFP^+^ mMDSCs ^(^green) were visible under two-photon confocal microscope. **I** Percentage of the area showing tdTomato+ cells per field in the intravital skull BM cavity from AA mice and mMDSC-treated mice on day14. Data were presented as the mean ± SD. *N* = 5 in each group. ***p < 0.001. FACs analyses of representative dot plot (**J**) and mean frequencies (**K**) of intra-BM CD4^+^ T cells and CD8^+^ T cells in AA mice and mMDSC-treated mice on day14. Data were presented as the mean ± SD. *N* = 6 in each group. *P < 0.05, **P < 0.01.

We thus performed multi-injection of mMDSCs (3 × 10^6^ per mouse) in AA mice at days 0,3,7 and 10 (Fig. 5A), showing significant benefit in prolonging OS compared to AA mice (Fig. 5B). In mMDSC-treated mice, the blood cell counts and the numbers of nucleated cells in BM tended to be recovered compared to untreated AA mice (Fig. 5C, D). Consistently, lower IFN-γ concentration was observed in the BM of mMDSCs-treated mice, with a notable rescue of hematopoietic colony formation of BM cells (Fig. 5E, F and Supplementary Fig. 3G).

To further investigate the immune-suppressive effect of mMDSCs in vivo, we injected tdTomato^+^ LN cells into sublethally irradiated CbyB6F1 mice in the presence or absence of GFP^+^ mMDSCs. Massive tdTomato^+^ LN cells were observed infiltrating into AA-BM (Fig. 5G–I). The BM structure of mMDSCs-treated mice showed fewer adipocytes and more cellularity than AA mice (Fig. 5G). After multi-injection of mMDSCs, donor-derived GFP-expressing mMDSCs accounted for 10% in the BM of AA mice on day 14, where intra-BM tdTomato^+^ LN cells were reduced from 61.70 ± 4.36 to 11.41 ± 1.38% (Supplementary Fig. 3H–J). Compared to AA mice, the frequencies of both intra-BM CD4^+^ T cells and intra-BM CD8^+^ T cells were diminished with the increasing GFP^+^ mMDSCs infiltration (Fig. 5J, K and Supplementary Fig. 3I).

### Monocytic MDSCs restored immune disorders in AA-BM

**Fig. 6:**
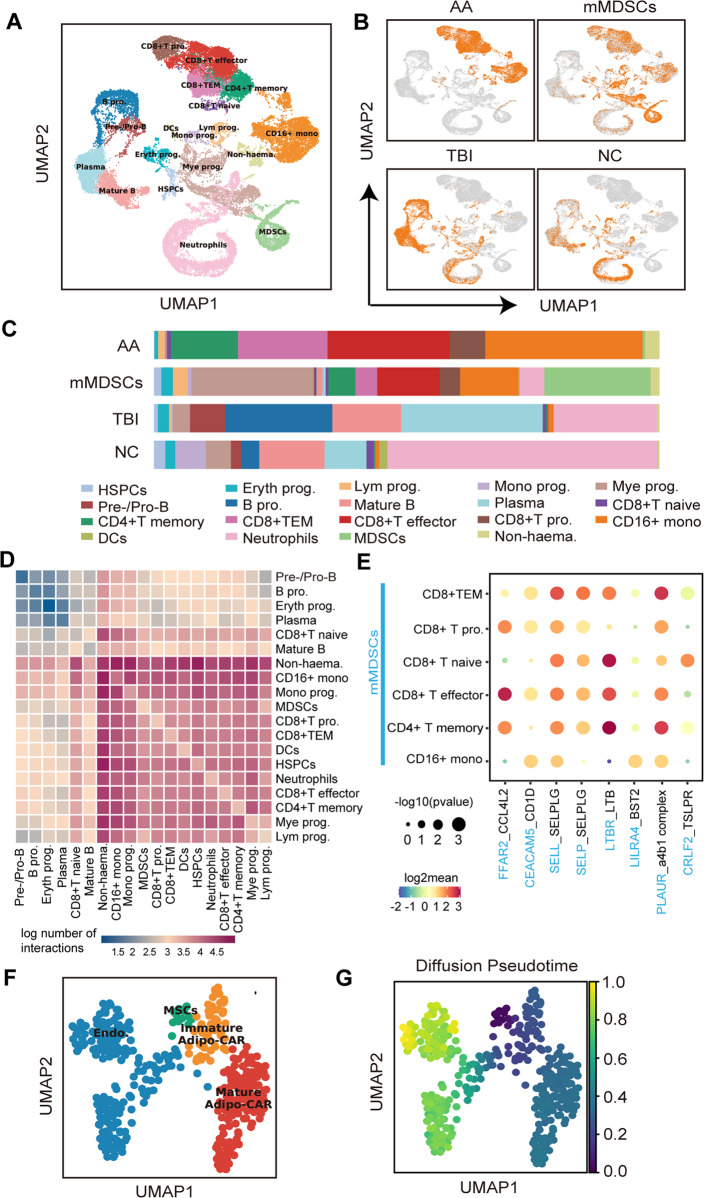
Identification of the relationship between different BM population by scRNAseq. **A** UMAP embedding a total of 34,308 BM cells which were pooled from AA mice (*n* = 2), mMDSCs-treated mice (*n* = 2), TBI mice (*n* = 2), and NC mice (*n* = 2) (Eryth prog.: erythroid progenitors; Lym prog.: lymphomyeloid progenitors; Mono prog.: monocyte progenitors; Mye prog.: myeloid progenitors; HSPCs: hematopoietic stem/progenitor cells; MDSCs: myeloid-derived suppressor cells; B pro.: B-proliferation; CD8^+^ TEM: CD8^+^ effector memory T cell, CD8^+^ pro.: CD8^+^ proliferating cell, Non-haema: non-hematopoietic cells) **B** UMAP independently embedding BM cells from AA mice (*n* = 2), mMDSCs-treated mice (*n* = 2), TBI mice (*n* = 2) and NC mice (*n* = 2). **C** The proportion of different population in BM cells from AA mice, mMDSCs-treated mice, TBI mice, and NC. **D** Heatmap depicting the log number of all possible interactions between various populations. **E** Dot plot depicting selected interactions between MDSCs and various immune response cellular populations in the BM microenvironment. **F** UMAP embedding different non-hematopoietic cell populations (Endo: endothelial cells, MSCs: mesenchymal stem cells, CAR: Cxcl12-abundant reticular cells). **G** UMAP embedding non-hematopoietic cells colored by the distance of diffusion pseudotime.

In a scRNA-seq on the BM cells of TBI, AA, and mMDSC-treated mice, nineteen cell clusters were defined by the characteristic gene expression patterns (Fig. 6A, Supplementary Figs. 4 and 5A). Notably, specific T cell-enriched clusters and CD16^+^ monocyte population distinctly appeared in AA mice but not in TBI and NC mice (Fig. 6B, C). After mMDSCs treatment, the progenitors of myeloid, erythroid, lymphoid and hematopoietic stem/progenitor cells (HSPCs) were reconstituted with the increased MDSCs cluster and decreased T cell and CD16^+^ monocytic clusters (Fig. 6B, C and Supplementary Fig. 5B). Among them, CD4^+^ T memory cells (TM), CD8^+^ effector memory T cells (TEM) and CD8^+^ T effector (TE) cells showed more significantly than CD8^+^ naïve and proliferating (pro.) T cells (Supplementary Fig. 5C). Additionally, the ratios of CD4^+^TM/CD8^+^ TEM cells in AA mice were lower than those in TBI mice but were partially recovered in mMDSCs-treated mice (Supplementary Fig. 5D). This result was consistent with the FACs analysis of the restored ratio of CD4^+^/CD8^+^ T cells (Fig. 5J, K).

By using SCENIC [13], ten transcription factor regulatory networks (regulons), Hand2, Pbx1, Sox9, Jdp2, Lbx2, Erg, Rxrg, Klf5, Arid3a, and Mafg activated in MDSCs compared to other cells (Supplementary Fig. 5E). We then explored the gene expression pattern of T cells in AA mice and mMDSCs-treated mice (Supplementary Table 3). The signature genes associated with T cell activation, proliferation, and differentiation were significantly triggered in AA mice (Supplementary Fig. 5F). Cytokines profile showed that IFN-γ, perforin, Granzyme B, and IL-2 were decreased in mMDSCs-treated mice (Supplementary Fig. 5G), indicating that mMDSCs transfusion helps inhibit AA-related pro-inflammatory status and restore the immune imbalance within BM.

By interpreting the expression of multi-subunit ligand-receptor complexes [14], we found that both CD8^+^ T cells and MDSCs were interacted with non-hematopoietic cells, HSPCs and CD4^+^ memory T cells more than with naïve T cells, plasma cells, and B cells. Not surprisingly, MDSCs and CD8^+^ T cells were highly linked with each other in the interaction heatmap (Fig. 6D). In our analysis, MDSCs might inhibit the inflammatory activity of T cells via the CD1a, BST2, CCL4L2, and SELPLG axis (Fig. 6E), whose functions were previously implicated in controlling T-cell’s proliferation, activation, trafficking and homing of CD8+ T cells [15–18].

To further dissect the relationship between different non-hematopoietic populations, we performed the pseudo-time analysis and found that BM-MSCs mainly differentiated into Adipo-Cxcl12-abundant reticular (CAR) cells and endothelial cells (Fig. 6F, G and Supplementary Fig. 6). The proportions of both immature and mature Adipo-CAR cells were significantly increased in AA mice compared to TBI mice. Notably, the immature adipo-CAR cells with stronger adipogenic differentiation potential were decreased after mMDSC treatment (Supplementary Fig. 5H).

### Monocytic MDSCs inhibited T cell-mediated MSC adipogenesis both in vitro and in vivo

**Fig. 7:**
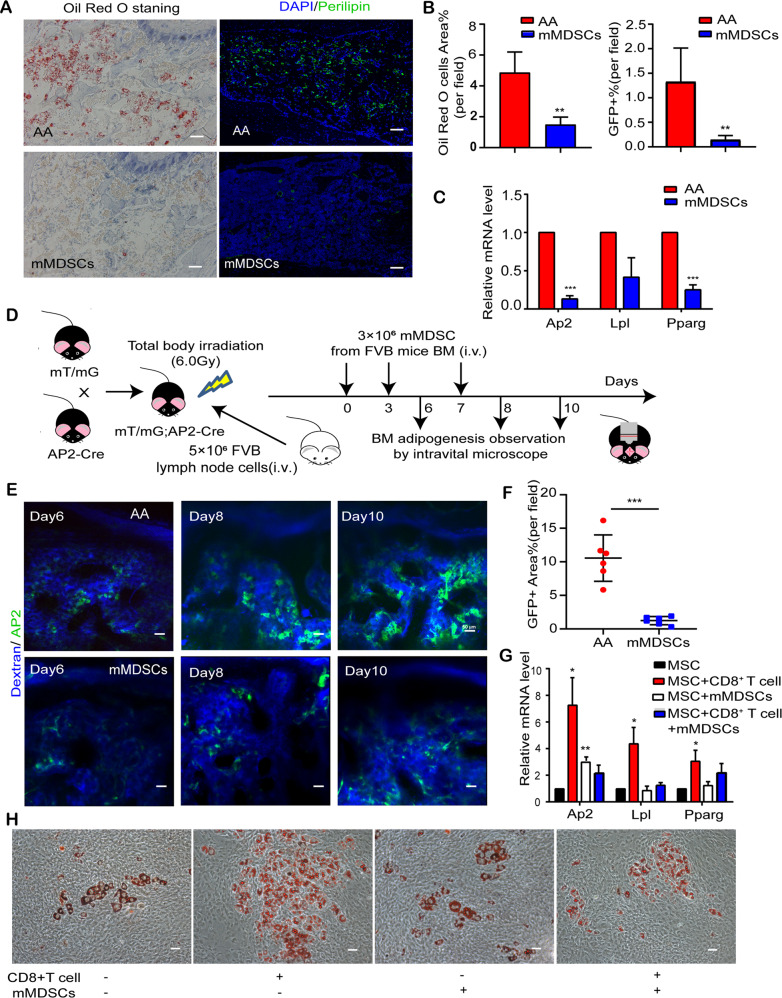
Monocytic MDSCs inhibited T cell-enhanced MSCs’ adipogenic differentiation in vitro and in vivo. **A** Representative images of femur BM stained with Oil Red O (left) and perilipin (right) from AA mice and MDSCs treated mice on day 14. Nuclear cells were stained by DAPI (blue) in the right panel. Scale bar:100 μm. **B** Percentage of the area showing positive Oil Red O staining (left) and GFP^+^ cells (right) in femur tissue of AA mice and mMDSC-treated mice on day 14. Data were presented as the mean ± SD. *N* = 4 in each group. **P < 0.01. **C** Quantitative RT-PCR analyses of *Ap2, Lpl,* and *Pparg* mRNA level in the BM of AA mice and MDSC-treated mice on day 14. Data represent mean±SEM in three independent experiments. *N* = 5 in each group. ***P < 0.001. **D** Schematic outline of adoptive mMDSCs treatment in AA mice to observe BM adipogenesis intravitally in vivo. Pre-irradiated (6.0 Gy) mT/mG;AP2-Cre mice (B6 background) were injected with 5 × 10^6^ LN cells derived from FVB mice. For mMDSCs treatment, 3 × 10^6^ mMDSCs sorted from FVB mice BM were injected on day 0, 3, and 7. Intravital microscopic observations were performed on day 6, 8, and 10. **E** Representative in vivo image of skull BM from mT/mG; AP2-Cre AA mice and mMDSC-treated mice. Vessels were stained in blue by tail vein injection of dextran-CY5 while AP2-GFP^+^ cells were visualized in green. Scale bar:50 μm. **F** Percentage of the area showing GFP^+^ T cells per field in the image of intravital skull BM cavity from AA mice and mMDSC-treated mice on day10. Data were presented as the mean ± SD. *N* = 6 in each group. ***P < 0.001. **G** Expressions of *Ap2, Lpl,* and *Pparg* in murine MSCs post-adipogenic differentiation were assessed by qRT-PCR, in the presence or absence of CD8^+^ T cells and mMDSCs. Data represent mean±SEM in three independent experiments *P < 0.05, ** *P* < 0.01. **H** Representative image of Oil Red O staining of murine MSCs post-adipogenic differentiation, in the presence or absence of conA activated CD8^+^ T cells and mMDSCs in vitro. Scale bar:50 μm.

Next, we detected the function of mMDSCs on BM-MSCs’ adipo-osteogenic differentiation. Compared with AA mice, intra-BM hypoplasia was relieved in company with less Oil Red O staining or perilipin-expressing fatty space in mMDSCs multi-treated BM (Fig. 7A, B). Inhibition of intra-BM adipogenesis in mMDSCs multi-transfused mice was also demonstrated by the downregulation of *Ap2*, *Lpl,* and *Pparg* (Fig. 7C). To detect the sequential BM adipogenic progression in AA microenvironment, we monitored the fluorescence expression of AP2 (Fabp4)-Cre×mT/mG reporting mice at different time points of T cells and mMDSCs transfusion by an in vivo fluorescent microscopy (Fig. 7D). As a membrane transport protein primarily expressing in adipocytes, AP2-Cre mice drove membrane GFP expression in adipocytes while all other cells were labeled with membrane tomato (Supplementary Fig. 7A). As shown in Fig. 7E, F, GFP-expressing AP2^+^ adipocytes accumulated in a time-dependent manner after T cell infusion. Coincident with our in vitro findings, mMDSCs multi-transfused AA mice showed decreased GFP^+^ adipocytes within BM cavity.

To confirm the regulating role of mMDSCs on BM adipogenesis in vitro, we additionally investigated the adipogenic feature of BM-MSCs by Oil Red O staining and gene expression of *Ap2, Lpl, and Pparg*, in the culture system with activated CD8^+^ T cells, mMDSCs or both. BM-MSCs cultured with CD8^+^ T cells had more expression of adipogenic genes and increasingly stained by Oil Red O while cocultured with mMDSCs did not (Fig. 7G, H). Indeed, the polarized T cell-triggered adipogenic differentiation was restored when mMDSCs were adding in the culture system of T cells and MSCs, showing less Oil Red O-staining and lower *Ap2, Lpl, and Pparg* expression on adipogenic-stimulated BM-MSCs (Fig. 7G, H and Supplementary Fig. 7B).

## Discussion

AA is a life-threatening disease being characterized by T-cell mediated BM hypoplasia and pancytopenia. The aberrant T cell immunity in AA consequently breaks up the intra-BM immune balance and results in hematopoietic dysfunction [19, 20]. Dissecting the relationship between T cell over-activation and hematopoiesis will help to explore the pathogenic mechanism and modify the therapeutic strategy to this disease entity.

In BM aspiration and biopsy samples from the patients who were diagnosed as AA, massive activated lymphocytes infiltration and adipocytes accumulation were observed as previously described [21]. Additionally, we found that the percentage of immune-modulatory MDSCs in AA mice’s BM were reduced. All these phenomena indicated an abnormally polarized BM adipogenic microenvironment and immune disorder under AA circumstance.

Various cellular and protein components in BM have been indicated to orchestrate the dynamic balance of HSPCs during the states of quiescence, self-renewal and differentiation [22]. BM-MSCs, the multipotential nonhematopoietic progenitors [23–28], regulate normal hematopoiesis by providing physical support to HSPC and secreting hematopoietic cytokines, which are termed together as “hematopoietic niche” [2, 29, 30]. The balanced adipo-osteogenic niche provided by BM-MSCs has been reported to contribute to HSPC maintenance and differentiation [31]. However, the pathologic role of the imbalanced adipo-osteogenic niche in AA remains largely unknown.

In our study, IFN-γ ‘licensed’ BM-MSCs were found to alter their differentiation potential toward adipocytes, indicating that not only HSPCs but also BM microenvironment are attacked by aberrant T cellular immunity. Consequently, polarized BM-MSCs adipogenic potential in the over-activated CD8^+^ T cell circumstance demonstrated that BM cavities might not be ‘passively’ filled with lipid droplets during the AA process. The over-activated CD8^+^ T cell-driven adipogenesis could be the fundamental factor for the fatty AA-BM, yet the function of intra-BM adipocytes is in debate [32–34]. As shown in present and other studies [35, 36], intra-BM adipocytes are located in the perivascular niche where the endothelial and Lepr-expressing stromal cells reside, as the specific space contributing to HSC maintenance [29, 37]. The overgrowth of BM-MSCs-derived adipocytes in the lumen wall may result in sinusoid collapse to exclude from blood flow, thus hampering normal hematopoiesis. Interestingly, Pparγ antagonist has been used to treat AA mice model [9], suggesting that the therapies targeting BM adipogenesis will be one promising tool in treating AA.

Among cellular immune-regulating populations, myeloid cells have been identified as important modulating cells in BM microenvironment. Physiologically, monocytes/macrophages regulate HPSC mobilization and promote stromal cells secreting CXCL-12 [38]. In some pathological conditions including tumors, infection, and autoimmune disorders, the immature myeloid cells known as MDSCs, are found to stop differentiating and expanding partially, followed by infiltrating into the inflammatory environment, to contribute to negative immune regulating response [39, 40]. Thus, this myeloid population has been thought to be a cellular therapeutic source to treat GVHD [41–43], autoimmune hepatitis [44, 45] as well as inflammatory bowel disease [46, 47].

In the present study, the decreased percentage of MDSCs in murine BM indicated that the intra-BM immune balance is shifted to CD8^+^ T-cell mediated immune over-reaction, among which mMDSCs were more important than gMDSCs to inhibit T-cell proliferation. Furthermore, in the activated regulons in MDSCs, Sox9, Klf5, and Mafg were reported to regulate the transcription of iNOS during inflammatory process [48–50]. With the view of the translational application, investigation on the role of iNOS pathway in the inhibition of T cells in vivo is required in future study.

In AA pathogenesis, acquired mutation, altering proliferation, susceptibility to apoptosis, or activated/inactivated signaling pathways can be the initiated response to lead lineage persistence, tissue destruction, or exposure of new antigens, which subsequently induces recruitment of immune cells [51]. Previous research showed that AA-linked hematopoietic hierarchy is associated with a selective repression at the lineage-committed progenitor level [52]. Over-activated T cells mediate MDSC apoptosis via Fas-FasL pathway [53], which results in decrease of MDSC population and intra-BM immune imbalance.

The sequencing BM transcriptomes at a single-cell level helped to track the cellular and molecular dynamics supporting the remission of BMF after mMDSCs therapy. The closer relationship between BM-MSCs and adipo-CAR cells under T-cell mediated circumstance jigsawes the important piece in the microenvironment puzzle of AA-BM. Consequently, adoptive multi-transfusion of mMDSCs reduced the intra-BM frequencies of CD4^+^ and CD8^+^ T lymphocyte, decreased intra-BM IFN-γ concentration, and ameliorated pancytopenia as well as BM destruction of AA mice. Along with the hematopoietic recovery post-mMDSCs transfusion, intra-BM adipogenic polarization was restored both in vivo and in vitro, indicating that mMDSCs as a functional population in BM niche maintain the intra-BM immune balance and play an important role in supporting normal hematopoiesis. In other words, mMDSCs might be considered as a potential agent for IST but not myeloid progenitors compensating for AA BM. Durable therapeutic effect of mMDSCs treatment on AA survival needs to be determined in future.

**Fig. 8:**
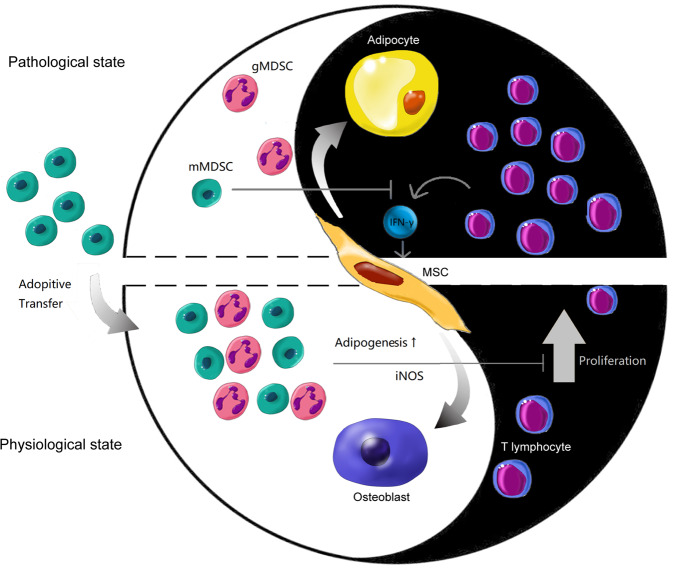
Effect of Yin (intra-BM immune over-activation) and Yang (intra-BM immune-modulation) on MSC differentiation within BM microenvironment. In AA BM, T cells (especially CD8+ T cells) are over-activated by AA-relating irritators, resulting in a Th1 immune response and increasing secretion of IFN-γ. Inflammatory IFN-γ licensed BM-MSCs shift their osteo-adipogenic differentiating potential towards adipogenic lineage which barely be able to support hematopoiesis. Reconstitution of the balance in BM microenvironment by immune modulating cells consequently improves microenvironment which is required for normal hematopoiesis.

In conclusion, hematopoiesis is a dynamic process requiring the excellent rapport between HSPCs and the surrounding microenvironment, which consists of stroma, cytokines, and immune regulators. Intra-BM immune balance is taken as one of the environmental parts seesawing as activating and suppressive ends. By contacting AA-related pathogenic irritation, the over-activated CD8^+^ T cells lodge into BM and commit to the immune attack on hematopoietic tissues. The multi-differential potential of BM-MSCs is also polarized towards adipogenesis by the stimulation of hyperactivated cytotoxic T cells and relating cytokines, which makes the BM microenvironment even barely support hematopoiesis. Adoptive transfusion of mMDSCs might be a novel immune-regulating strategy to treat AA, accounting for not only restoring the intra-BM immune balance but also improving stroma’s multi-differentiating microenvironment (Fig. 8).

## Materials and methods

### Patients

Six BM samples from AA patients were studied, whereas those from lymphoma patients without BM involvement were used as BM controls. The protocol of this study was approved by the Institution Review Board of Huashan Hospital, Fudan University (No. 2017–278).

### Animals

C57BL6 (B6), Balb/c, and FVB/N (FVB) mice were obtained from Shanghai SLACCAS Animal Laboratory (Shanghai, China). B6.STOCK-Tg(CAG-EGFP)/Nju (GFP), B6.Cg-Tg(Fabp4-cre)1Rev/J (Fabp4-Cre), B6.129(Cg)-Gt(ROSA)26^Sortm4(ACTB-tdTomato-EGFP)/Nju^ (mT/mG) were obtained from Model Animal Research Center of Nanjing University. Hybrid CbyB6F1 mice were bred and maintained in Fudan University under standard care and nutrition. The age-matched male mice (8–12 weeks old) were used in the experiments. The animal experiments were carried out according to the regulation of Experimental Animal Ethics Committee of Fudan University (No. 2016-02-HSYY-CT-01). Detailed procedures of generating AA mice and mMDSCs-treated AA mice were indicated in Supplemental Methods.

### Flow cytometry analysis and cytometric bead array

Up to 1 × 10^6^ cells were stained with antibodies at 4 °C for 20 min. For intra-cellular staining, murine cells from BM or spleen were incubated with fixation/permeabilization working buffer (eBioscience, Vienna, Austria) for 1 h before staining. The applied antibodies can be found in Supplementary Table 1. Flow cytometry analysis was performed on Cyan (Beckman Coulter, Brea, CA USA).

To perform intra-BM cytokine analysis, total BM from femurs were flushed out with 0.5 ml PBS. The cytokines in murine BM were determined by Th1/Th2 cytometric bead array kits (BD Pharmingen, San Diego, CA, USA) and detected on Arial II (BD Biosciences, San Diego, CA, USA). Data were analyzed with FCAP Array (BD Pharmingen).

### Adipogenic or osteogenic differentiation of BM-MSCs

BM-MSCs were derived and induced into adipocytic or osteogenic differentiation. Oil Red O solution and alkaline phosphatase staining were used to detect the adipocytic or osteogenic differentiation of MSCs, respectively. Detailed procedures were indicated in Supplementary Methods.

### Nitrite production (NO) assay

T lymphocytes were cultured alone or together with CD11b^+^Ly6C^+^Ly6G^-^ monocytic MDSCs (mMDSCs) or CD11b^+^Ly6C^-^Ly6G^+^ granulocytic MDSCs (gMDSCs) for 72 h. The concentrations of NO in the supernatant were determined with modified Griess reagent by nitrite detection kit according to the manufacturer’s instructions (Beyotime, Shanghai, China). The absorbance was measured at 540 nm by Microplate Eon (BioTek, Winooski, VT, USA).

### Adoptive transfusion of mMDSCs in AA mice

To assess the therapeutic function of mMDSCs in AA, mMDSCs sorted from B6-BM were intravenously injected in AA mice at the number of 5 × 10^6^ cells/mouse of single injection on day 0, or 3 × 10^6^ cells/mouse of multi-injection on day 0, 3, 7, and 10. The same amount of mMDSCs sorted from murine FVB BM were administrated to mT/mG; AP2-Cre AA mice on day 0, 3, and 7. The blood counts were determined on automated hematology analyzer (Mindray, Shenzhen, China).

### Single-cell capture and RNA-sequencing (scRNA-seq)

BM cells were collected from AA mice, mMDSC-treated AA mice, TBI and normal control (NC) mice. The experimental procedures were followed with the established techniques using the Chromium Single Cell 3’ Library V3 kit (10 × Genomics). Briefly, BM cells were encapsulated into droplets and loaded into the Chromium instrument (10 × Genomics). The resulted barcoded cDNAs were used to construct libraries with the Single Cell 3’ Library. Raw sequence data were converted into FASTQs by Illumina bcl2fastq software, which were aligned to the Mus musculus genome (GRCm38) using the CellRanger v3.0.1 pipeline (10 × Genomics) according to the manufacturer’s instructions. Detailed procedures were indicated in Supplemental Methods.

### Histology and immunofluorescence detection

For immunofluorescence labeling, the deparaffinated section was stained with anti-perilipin (1:100, Abcam, Cambridge, MA, USA), anti-GFP (1:200, Abcam), and 4′,6-Diamidino-2-Phenylindole (DAPI, 0.5 µg/mL, Invitrogen, Carlsbad, CA, USA). A confocal microscope (Leica SP8 MP, Wetzlar, Germany) was used to detect fluorescence images. Quantification of fluorescence signal was determined by ImageJ software (USA).

### Intravital microscopy

In vivo BM imaging of the skull bone in live animals was described previously [54]. Fifty μl of Cy5-conjugated dextran (8 mg/ml, Yuanye, Shanghai, China) was injected via the tail vein to stain the blood vessel. By exposing the skull bone, fluorescence images were acquired by using a two-photon confocal microscope (Leica SP8 MP).

### Statistical analysis

All experiments were performed in 3–5 replicates and repeated independently at least three times. The data were presented as mean ± SD. Two-tailed student t-test or Mann–Whitney U test was used for comparison between two groups. Differences in survival rates were determined by log-rank statistics analysis. Difference was considered statistically significant at *P < 0.05, **P < 0.01, ***P < 0.001.

## Supplementary information

Supplemental material

Supplemental Table1

Supplemental Table2

Supplemental Table 3

Reproducibility Checklist

## Data Availability

The datasets analyzed during the current study are available through the accession number CRA007234 in the National Genomics Data Center (NGDC) repository.
