# Supplementary material for: Monocytic myeloid-derived suppressive cells mitigate over-adipogenesis of bone marrow microenvironment in aplastic anemia by inhibiting CD8+ T cells
Source: Cell Death Dis. 2022 Jul 18;13(7):620. doi: 10.1038/s41419-022-05080-5 (PMC9293984; doi:10.1038/s41419-022-05080-5)
Supplement: Supplementary file 1 — Supplemental material [file 41419_2022_5080_MOESM1_ESM.docx]

**Supplemental Materials and Methods**

**Generation of aplastic anemia (AA) mice**

The procedure of murine AA induction was previously reported^1-2^. In brief, 5×10^6^ lymphocytes (lymph node cells, CD4^+^ or CD8^+^ T cells) were isolated from B6 or GFP donors and intravenously injected into sex-matched CbyB6F1 recipient, which were pre-irradiated with 5.0 Gy of total body irradiation (TBI) 2-4 hours ago. Similarly, the same amount of lymph node (LN) cells from FVB mice were infused into 6.0 Gy pre-irradiated B6 mice^3^.

**Isolation of different T cell populations and myeloid-derived suppressive cell (MDSC) populations**

LN cells from B6 donor mice were grinded through a 40μm nylon cell strainer (Falcon, USA), washed and stained with antibodies against CD4 or CD8. Different populations of T lymphocytes (CD4^+^ or CD8^+^ T cells) were sorted by fluorescence-activated cell sorting (FACS) (Arial II, BD Biosciences).

Bone marrow (BM) cells from CbyB6F1 AA mice or B6 mice were obtained and stained with antibodies against CD11b, Ly6G and Ly6C. Monocytic-MDSCs (mMDSC, CD11b^+^Ly6C^+^Ly6G^-^) and granulocytic-MDSCs (gMDSCs, CD11b^+^Ly6C^-^Ly6G^+^) were sorted by FACS (Arial II, BD Biosciences).

**Hematopoietic colony formation**

BM cells were flushed from the femurs of mice and plated in triplicate cultures (35 mm culture dishes) at a final plating concentration of 3 x 10^4^ cells/dish using MethoCult™ GF M3434 medium (StemCell Technologies, Vancouver, BC, Canada). The formation of hematopoietic colony-forming units (CFUs), including colony-forming unit-erythroid (CFU-E), burst-forming unit-erythroid (BFU-E), colony-forming unit-granulocyte, macrophage (CFU-GM), colony-forming unit-granulocyte, erythroid, macrophage, megakaryocyte (CFU-GEMM) was counted on day 14 in a humidified 5% CO_2_/37°C environment.

**Culture of BM-MSCs**

To isolate murine BM-MSCs, mice were sacrificed and BM cells from femurs and tibias were flushed out into 10 cm^2^ culture dish with L-DMEM (Gibco, Waltham, MA, USA) containing 10% fetal bovine serum (Gibco), 100 IU/ml penicillin, 100 mg/ml streptomycin (Gibco) and 2 mM L-glutamine (Gibco). Media were changed every 3 days.

For culturing human BM-MSCs, a total 3mL BM samples were collected from AA patients or controls. BM mononuclear cells were isolated by Ficoll-Paque (Cytiva, Marlborough, MA，USA) and seeded in 1cm^2^ culture dish with medium mentioned above. Non-adherent cells were discarded after 48h. Media were replaced every 3 days and adhered cells were trypsinized and replated until 80%-90% confluence.

**Induction of adipogenic or osteogenic differentiation of BM-MSCs**

Adipogenic and osteogenic differentiation were performed as previously described^4^. Briefly, after MSCs were plated at a density of 3×10^4^ cells per well of 24-well plates in the presence or absence of IFN-γ (10ng/mL), TNF-α (5ng/mL, Peprotech, Cranbury, NJ, USA), ConA (5 μg/mL，Sigma) activated CD3^+^ or CD8^+^ T cells (3×10^5^ per well), and mMDSCs (3×10^5^ per well) for 72h, the media were replaced with adipogenic differentiation medium or osteogenic differentiation medium (Cyagen, Suzhou, China). On day 12 of culture, these cells were used for further experiments.

For T cell stimulation, 72h before MSCs’ adipogenic induction, ConA activated CD3^+^ T cells were co-cultivated with MSCs in a 0.4μm pore size Transwell system, where both types of cells were plated in the same lower chamber forming direct contact, or T cells were loaded in upper chamber with MSCs in lower chamber showing non-direct contact.

**Quantitative real time-polymerase chain reaction (qRT-PCR)**

RNAs were extracted by TRIzol according to the manufacturer’s instruction (Thermo Fisher Scientific, Inc., Waltham, MA, USA) and were reversed transcribed to cDNA by using the Primer Script RT reagent Kit (Takara, Tokyo, Japan). RT-PCR reactions were run on a StepOnePlus Real time PCR System (Thermo Fisher Scientific, Inc.) by using SYBR Green PCR Master Mix (Takara). All data were normalized to the expression of β-actin. The primers used for qPCR are listed in Supplemental Table 2.

**Oil Red O Staining and Alkaline Phosphatase (AKP) staining**

Briefly, the cells or frozen tissue sections were fixed in 4% formaldehyde for 1h at room temperature. After washing with distilled water, the cells were stained with 0.21% Oil Red O in 100% isopropanol (Sigma) for 10 min. Pictures were captured using an Olympus BX53 microscope and quantified by ImageJ software. To calculate the intracellular lipid accumulation, the stained lipid droplets were dissolved with 100% isopropanol for 5min. Optical density was measured at 510 nm by the ELISA reader (Biotek).

For AKP staining, the cells were fixed in 4% formaldehyde for 1 h. After the cells were rinsed with distilled water, a 5-bromo-4-chloro-3-indolyl phosphate/nitroblue tetrazolium solution (Beyotime) was added to each well. The stained cells were washed and photographed.  Data was analyzed by ImageJ software (National Institutes of Health, USA).

**Proliferation and mixed lymphocyte reaction (MLA) of T cells**

For carboxyfluorescein diacetate succinimidyl ester (CFSE) labeling and MLA analysis, sampled T lymphocytes were resuspended with PBS at a concentration of 1×10^6^ cells/mL, and stained with 1μM APC-CFSE (ebioscience) for 10 minutes at 37°C, and washed with culture medium for 3 times. A total of 2×10^5^ CFSE-labeled T cells were stimulated with ConA (5μg/mL) in the absence or presence of 2×10^5^ mMDSCs, and incubated with murine G-CSF (400U/ml), GM-CSF (50U/ml) (PeproTech) for 72h. Cells were incubated with antibodies against CD3 or CD8 (Biolegend, San Diego, CA, USA) for 15min.

CFSE labelling curve of CD8^+^ T cells and loaded cell numbers per minute at the same in-flux flow rate were determined by FACs analyses on Cyan (Beckman).

**Sample collection of single-cell capture and RNA-seq (scRNA-seq)**

BM cells were flushed from the murine femurs with PBS, then passed through cell strainers and centrifuged at 400 g for 10 min. After the supernatant was removed, the pelleted cells were suspended in red blood cell lysis buffer (Miltenyi, Bergisch-Gladbach, Germany) and incubated on ice for 2 min to lyse red blood cells. After washing twice with PBS, the cell pellets were re-suspended.

**Single-cell 3′ mRNA sequencing**

A total of 34,308 pooled BM cells (n=8) were encapsulatingly separated into droplets, and libraries were constructed using the Single Cell 3' Library & Gel Bead Kit (10x Genomics) Chromium following the manufacturer's instructions^5^. The libraries were finally sequenced using an Illumina Novaseq6000.

**Single-cell RNA seq data processing and quality control**

Illumina sequencer base call files were converted into FASTQs using the Illumina bcl2fastq software. FASTQ files were aligned to the Mus musculus genome (GRCm38) using the CellRanger (version 3.1.0) pipeline according to the manufacturer's instructions. The initial gene expression matrix was then processed and analyzed by Scanpy (version 1.5.1)^6^. The cells were included with genes greater than 500 but less than 6000, the percentage of mitochondrial reads less than 15%, and the percentage of hemoglobin reads less than 2%.

**Integrated analysis, dimensionality reduction, clustering, and visualization**

After filtering, the expression matrix was normalized by the “scanpy.pp. normalize_total” function in the Scanpy package and in-transformed by the “scanpy.pp.log1p” function. Then we utilized Scanorama algorithm in Scanpy package to correct the combined dataset for technical batch effects ^7^. Clustering was performed using graph-based clustering and visualized by using Uniform Manifold Approximation and Projection (UMAP).

**Identification of cell types**

Cell types were manually assigned to the clusters by comparing the mean expression of known markers across cells in a cluster. Markers used to type cells included *Ptprc* (immunocytes), *Cd79a* (B cells), *Cd3g* (T cells), *Siglech* (dendritic cells), *S100a8* (neutrophils), *Csf1r* (monocytes), *Hbb-bt* (erythroid cells), *Kit* (hematopoietic stem/progenitor cells, HSPCs), *Flt3* (lymphomyeloid progenitors), *Gata1* (erythroid progenitors), *Itga2b* (megakaryocyte progenitors), *Mpo* (myeloid progenitors), *Ms4a2* (eosinophil/basophil progenitors), *Pdgfrah* (mesenchymal cells), *Cdh5* (endothelial cells), *Emcn* (sinusoidal endothelial cells), *Col1a1* (osteoblasts and fibroblasts), *Vcam1* (vascular endothelial cells).

**Cell-cell ligand-receptor interactions**

Cell-cell ligand-receptor interactions were inferred using the CellPhoneDB (version 2.0.0) method in Python ^8^. The lower cutoff for expression proportion of any ligand or receptor in a given cell type was set to 10%, and the number of permutation was set to 1000.

**Differential gene expression analysis**

To find markers that define cell clusters, we performed differential expression analysis on data by the “scanpy. tl.rank_genes_groups” function in the Scanpy package (wilcoxon test are calculated). The differential genes were subjected to Gene Ontology (GO) and KEGG analysis using the R package clusterProfiler (version 3.11)^9^.

**References**

1. Chen J, Lipovsky K, Ellison FM, Calado RT & Young NS. Bystander destruction of hematopoietic progenitor and stem cells in a mouse model of infusion-induced bone marrow failure. *Blood* **104**, 1671-1678 (2004).

2. Bloom ML, Wolk AG, Simon-Stoos KL, Bard JS, Chen J & Young NS. A mouse model of lymphocyte infusion-induced bone marrow failure. *Exp Hematol* **32**, 1163-1172 (2004).

3. Chen J, Desierto MJ, Feng X, Biancotto A & Young NS. Immune-mediated bone marrow failure in C57BL/6 mice. *Exp Hematol* **43**, 256-267 (2015).

4. Qu Y, Lin Q, Yuan Y, Sun Z, Li P & Wang F *et al*. Cyclosporin A inhibits adipogenic differentiation and regulates immunomodulatory functions of murine mesenchymal stem cells. *Biochem Bioph Res Co* **498**, 516-522 (2018).

5. Zheng GX, Terry JM, Belgrader P, Ryvkin P, Bent ZW & Wilson R *et al*. Massively parallel digital transcriptional profiling of single cells. *Nat Commun* **8**, 14049 (2017).

6. Wolf FA, Angerer P & Theis FJ. SCANPY: large-scale single-cell gene expression data analysis. *Genome Biol* **19**, 15 (2018).

7. Hie B, Bryson B & Berger B. Efficient integration of heterogeneous single-cell transcriptomes using Scanorama. *Nat Biotechnol* **37**, 685-691 (2019).

8. Efremova M, Vento-Tormo M, Teichmann SA & Vento-Tormo R. CellPhoneDB: inferring cell-cell communication from combined expression of multi-subunit ligand-receptor complexes. *Nat Protoc* **15**, 1484-1506 (2020).

9. Yu G, Wang LG, Han Y & He QY. clusterProfiler: an R package for comparing biological themes among gene clusters. *Omics* **16**, 284-287 (2012).

**Supplemental Figures and Legends**

**Supplemental Figure 1**


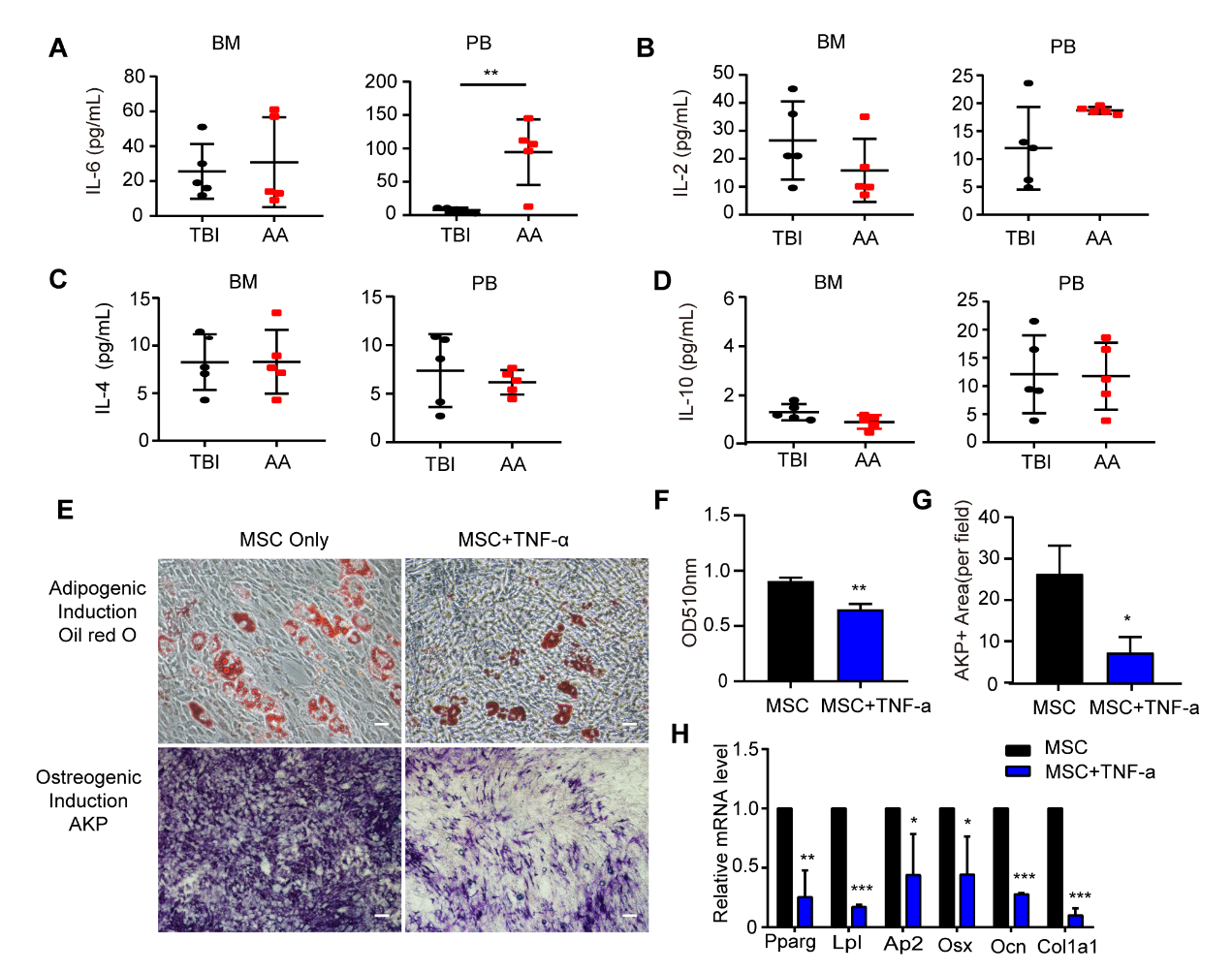


**Supplemental Figure 1. TNF-α hampered both adipogenic and osteoblastic differentiation of BM-MSCs.** The concentration of IL-2 (A), IL-6 (B), IL-4 (C) and IL-10 (D) in the serum and supernatant of BM flushing fluid from TBI mice or AA mice on day 14 were determined by CBA kit. Results were presented as the mean ± SD. N = 5 in each group. Data were pooled from three independent experiments. ***P*<0.01. (E) BM-MSCs were incubated with TNF-α (5ng/mL) for 72h before differentiation induction. After differentiation for 14 days or 7 days, adipogenic or osteogenic potential was assessed by Oil Red O staining and AKP staining respectively. Scale bar:50μm. (F) BM-MSCs were stained with Oil red O, destained with isopropanol, and the OD at 510 nm was determined. Results were presented as the mean ± SD. **P*<0.05, ***P*<0.01. Data were pooled from three independent experiments. (G) Quantification of AKP in the cells was determined by ImageJ software. Data were presented as the mean ± SD. Graph represents pooled data from three independent experiments. **P*<0.05. (H) Expressions of adipogenic markers (*Pparg*, *Lpl* and *Ap2*) and osteogenic markers (*Osx, Ocn and Col1a1*) of BM-MSCs were assessed by qRT-PCR after adipogenic or osteogenic induction in the presence or absence of TNF-α. Data represent mean±SEM in three independent experiments. **P*<0.05, ***P*<0.01, ****P*<0.001.

**Supplemental Figure 2**


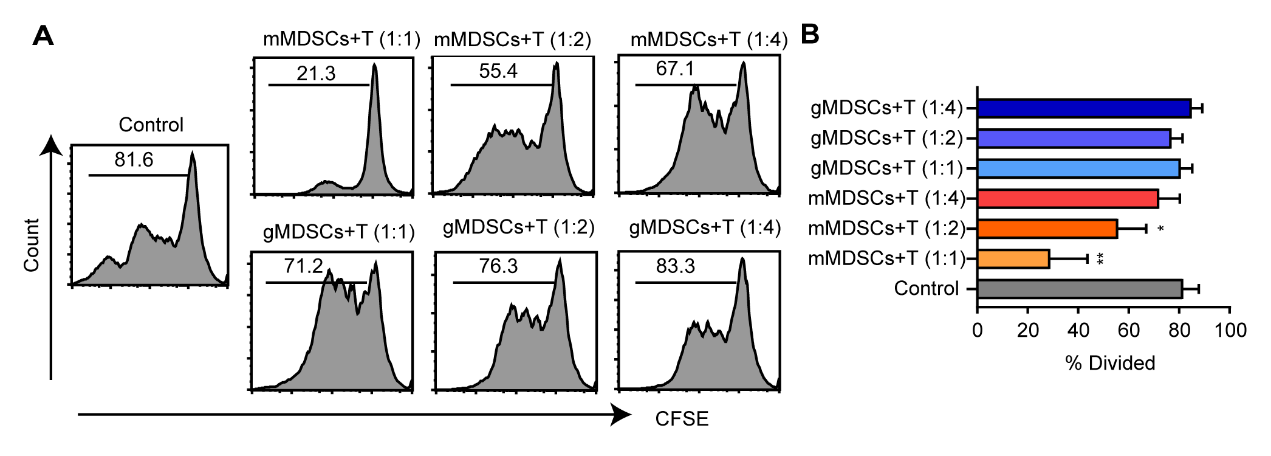


**Supplemental Figure 2. Monocytic MDSCs inhibited T cell proliferation and infiltration into AA-BM.** (A-B) CFSE-labelled CD8^+^ T cells were activated with ConA (5μg/mL) for 72h, and cocultured with mMDSC or gMDSC from AA-BM at the ratio of 1:1,1:2 and 1:4. (A)The inhibitory effect of MDSCs from AA-BM on CD8^+^ T cells proliferation. (B) Histograms represented the percentages of divided CD8^+^ T cells in the presence of ConA at different ratio of gMDSC/T cells and mMDSC/T cells. Data represent mean±SEM in three independent experiments. **P*<0.05, ***P*<0.01.

**Supplemental Figure 3**


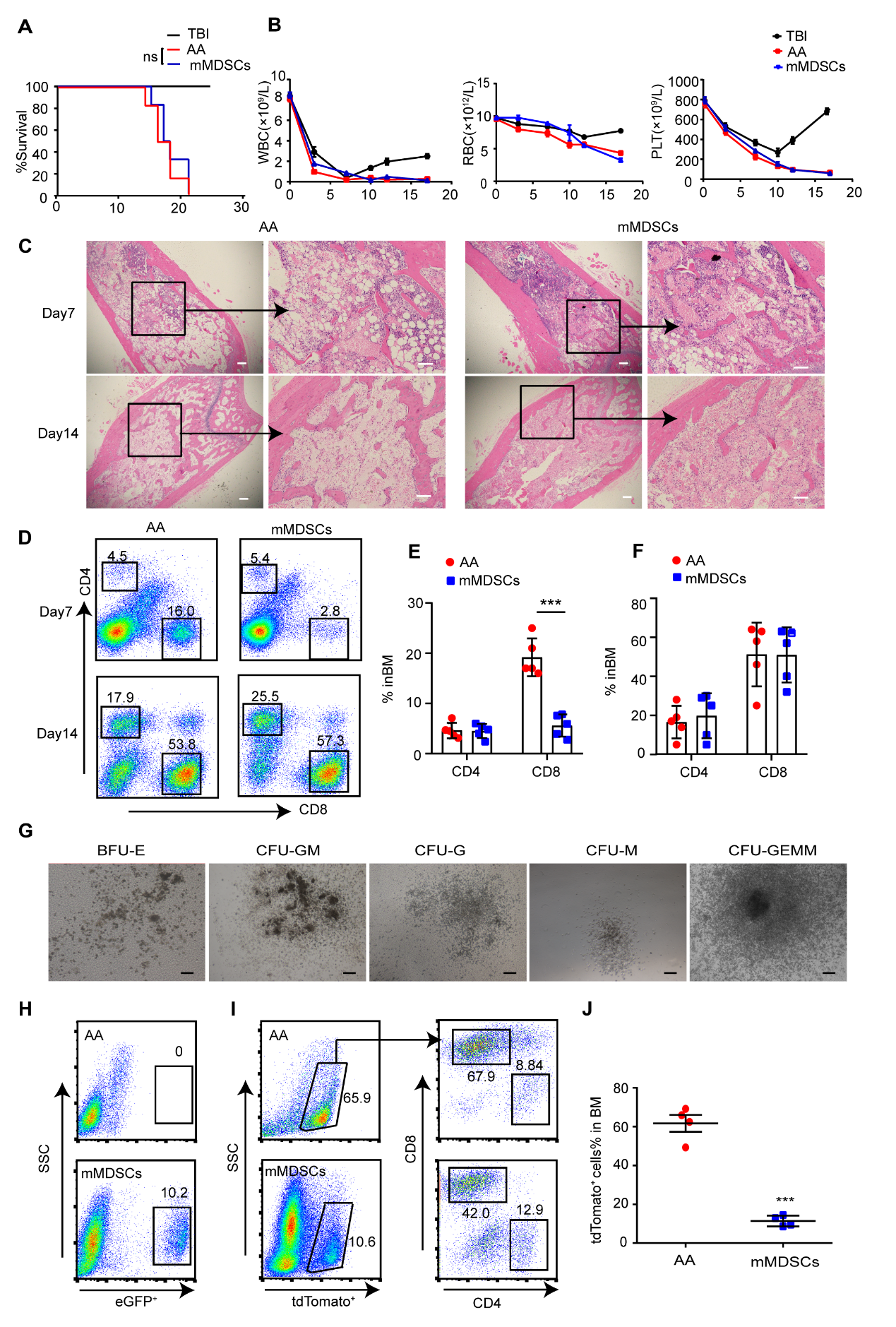


**Supplemental Figure 3. Monocytic MDSCs diminished T cell infiltration *in vivo* and suppressed T cell-triggered adipogenic differentiation *in vitro***. (A-B) Pre-irradiated CbyB6F1 mice were injected with 5×10^6^ B6 LN cells, with or without single dose of co-injection of 5×10^6^ mMDSCs at day 0. (A)The survival curves of TBI mice, AA mice and single dose mMDSCs-treated mice were determined. N=6 in each group from two independent experiments. (B) The blood counts of TBI mice, AA mice and single dose mMDSCs-treated mice were determined. Data were presented as the mean ± SD. N=4 in each group from two independent experiments. (C) H&E stainings of murine femurs in AA mice and single dose mMDSCs-treated mice on day7 and day14. Scale bar:100μm. (D) FACs analysis of intra-BM CD4^+^ T cells and CD8^+^ T cells in AA mice and single dose mMDSCs-treated mice on day7 and day14. Histograms represent the percentages of intra-BM CD4^+^ T cells and CD8^+^ T cells in AA mice and single dose mMDSCs-treated mice on day7 (E) and day14 (F). Data were presented as the mean ± SD. N=5 in each group from two independent experiments. ****P*<0.001. (G) Hematopoietic colonies at day 13 were pictured in bright field. scale bar: 50 μm. (H-J) Pre-irradiated CbyB6F1 mice were injected with 5×10^6^ tdTomato^+^ LN cells to induce AA, and treated with 3×10^6^ GFP-expressing mMDSCs on day 0, 3, 7, 10. The intra-BM frequencies of GFP+ mMDSCs (H) and donor-derived tdTomato^+^ cells in AA mice and mMDSCs-treated mice on day 14 were determined. (I) PE-Cy7-labelling CD4^+^tdTomato^+^ T cells and APC-labelling CD8^+^ tdTomato^+^ T cells were determined by FACs analysis. (J) Data showed the intra-BM frequencies of donor-derived tdTomato^+^ cells in AA mice and mMDSCs-treated mice on day 14. Data were presented as the mean ± SD. N=4 in each group from two independent experiments. ****P*<0.001.

**Supplemental Figure 4**


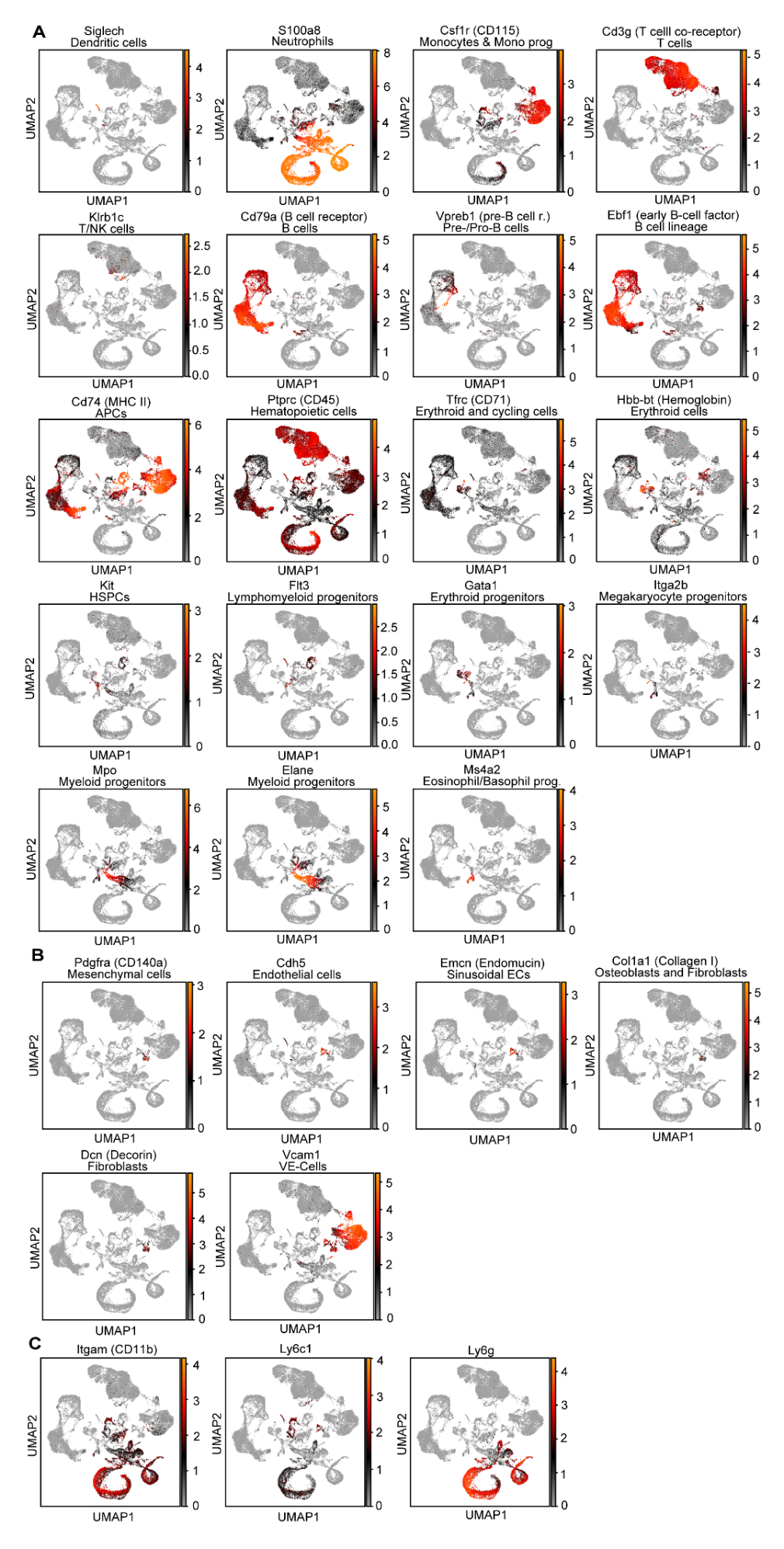


**Supplemental Figure 4. Feature plots demonstrating expression of specific genes among clusters to identify respective cell types.** UMAP embedding of the canonical hematopoietic (A), non-hematopoietic (B) and MDSCs (C) gene marker expression for cell-type annotation of the clusters described in Figure 6A.

**Supplemental Figure 5**

**
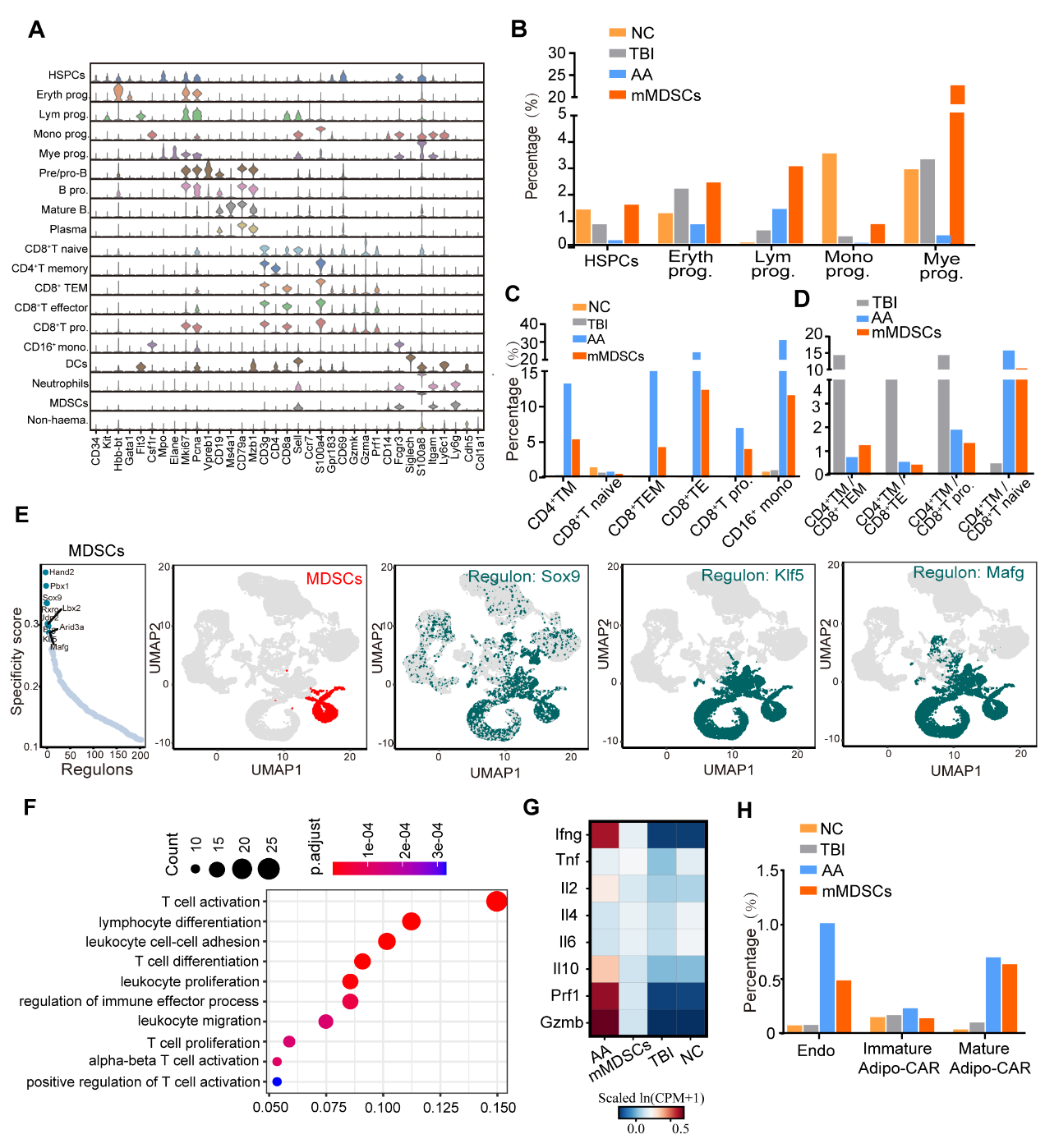
**

**Supplemental Figure 5.** **Identification of the relationship between different BM population by scRNAseq.** (A) Violin plots presenting the distribution of classical cell markers for each cell type. (B) Bar plots showing proportion of hematopoietic stem progenitor cells in different conditions. HSPCs: hematopoietic stem/progenitor cells; Eryth prog.: erythroid progenitors; Lym prog.: lymphomyeloid progenitors; Mono prog.: monocyte progenitors; Mye prog.: myeloid progenitors. (C) Bar plots showing proportion of T cells and monocytes in different conditions. CD4^+^ TM: CD4^+^ memory T cell, CD8^+^ TEM: CD8^+^ effector memory T cell, CD8^+^ TE: CD8^+^ effector T cell, CD8^+^ pro.: CD8^+^ proliferating cell. (D) Bar plots showing the ratio of CD4^+^ memory T cells to different CD8^+^ T cell clusters in each group. (E) UMAP showing the activity of transcription factors Sox9, Klf5, Mafg in MDSCs cluster. (F) Gene ontology term enrichment analysis based on the overexpressed genes in T cells (AA mice *vs* mMDSCs-treated mice). (G) Expression of IFNγ, TNFα, IL2, IL6, IL4, IL10, Perforin and Granzyme B in T cells from AA mice, mMDSCs-treated mice, TBI mice and NC. (H) Bar plots showing proportion of MSC and Adipo-CAR cells in different conditions.

**Supplemental Figure 6**

**
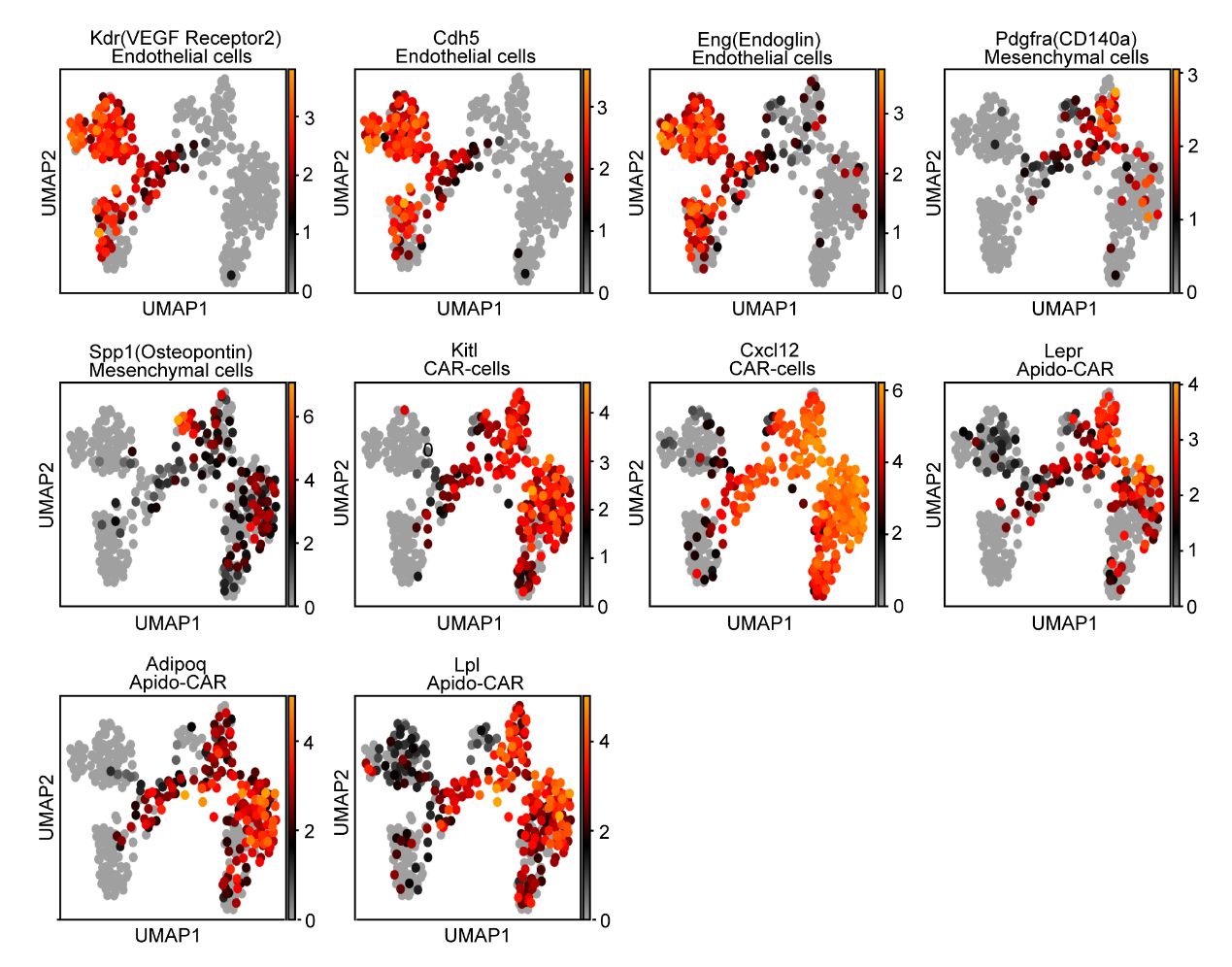
**

**Supplemental Figure 6. Feature plots demonstrating expression of specified genes among clusters to identify respective cell types.** UMAP embedding of marker genes expression for cell-type annotation of cells described in Figure 6F.

**Supplemental Figure 7**


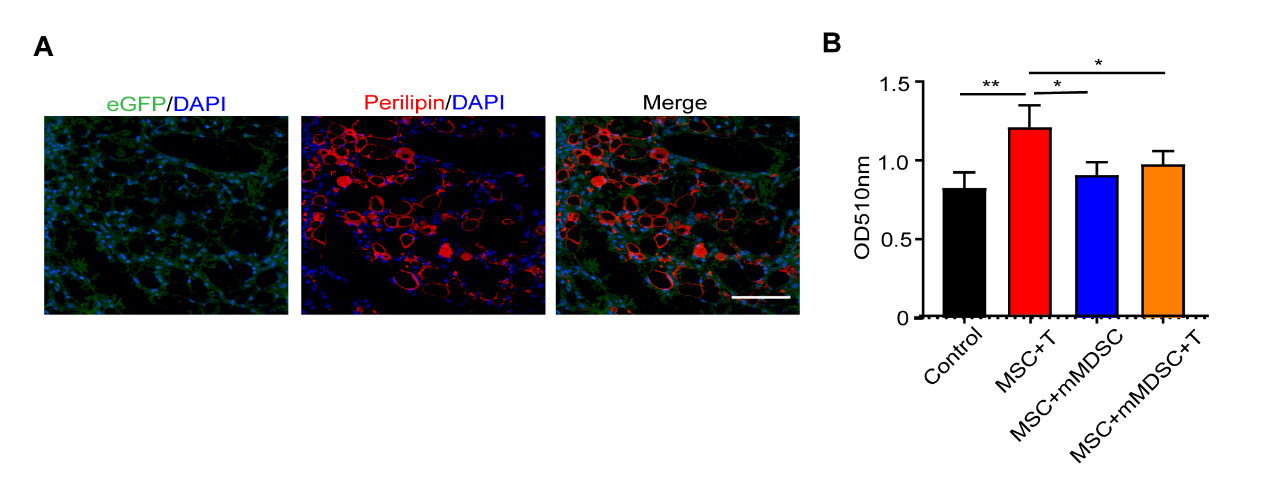


**Supplemental Figure 7. Monocytic MDSCs inhibited T cell-enhanced MSCs’ adipogenic differentiation.** (A) Staining of FITC-labeling rat against murine GFP (green), CY5-labelling rabbit against murine perilipin (red) and DAPI (blue) in mT/mG; AP2-cre murine femurs of AA mice was detected under confocal microscope. Scale bar:100μm. (B) Staining of Oil Red O was performed on BM-MSCs 12 days after adipogenic differentiation in the presence or absence of ConA activated CD8^+^ T cells and mMDSCs *in vitro*. OD value at 510 nm of Oil Red O staining was determined. Data represent mean±SEM in four independent experiments. **P*<0.05，***P*<0.01.
