# Supplementary material for: Monocytic myeloid-derived suppressive cells mitigate over-adipogenesis of bone marrow microenvironment in aplastic anemia by inhibiting CD8+ T cells
Source: Cell Death Dis. 2022 Jul 18;13(7):620. doi: 10.1038/s41419-022-05080-5 (PMC9293984; doi:10.1038/s41419-022-05080-5)
Supplement: Supplementary file 2 — Supplemental Table1 [file 41419_2022_5080_MOESM2_ESM.docx]

Supplemental Table 1. Antibodies for FACS analyses

| Antibodies | Clones | Source |
| --- | --- | --- |
| Anti-mouse Gr-1 PE | RB6-8C5 | Biolegend |
| Anti-mouse CD11b PerCP-Cy5.5 | M1/70 | Biolegend |
| Anti-mouse/human CD11b FTIC | M1/70 | Biolegend |
| Anti-mouse Ly6G PE | 1A8 | Biolegend |
| Anti-mouse Ly6C FITC | HK1.4 | Biolegend |
| Anti-mouse Ly6C APC | HK1.4 | Biolegend |
| Anti-mouse CD8a PE | 53-6.7 | Biolegend |
| Anti-mouse CD8a APC | 53-6.7 | Biolegend |
| Anti-mouse CD8a PE-Cy7 | 53-6.7 | Biolegend |
| Anti-mouse CD3 FITC | 17A2 | Biolegend |
| Anti-mouse CD4 PE-Cy7 | RM4-5 | Biolegend |
| Anti-mouse CD4 APC | RM4-5 | Biolegend |
| Anti-mouse CD25 APC | PC61.5 | eBioscience |
| Anti-mouse CD69 PE | FN50 | Biolegend |
