# Supplementary material for: Monocytic myeloid-derived suppressive cells mitigate over-adipogenesis of bone marrow microenvironment in aplastic anemia by inhibiting CD8+ T cells
Source: Cell Death Dis. 2022 Jul 18;13(7):620. doi: 10.1038/s41419-022-05080-5 (PMC9293984; doi:10.1038/s41419-022-05080-5)
Supplement: Supplementary file 3 — Supplemental Table2 [file 41419_2022_5080_MOESM3_ESM.docx]

Supplemental Table 2. Primers for RT-PCR analyses

| Species | Name | 5‘→3’ sequence | Name | 5‘→3’ sequence |
| --- | --- | --- | --- | --- |
| Mouse | β-actin-F | CATCCGTAAAGACCTCTATGCCAAC | β-actin-R | CACTGTGTTGGCATAGAGGTC |
|  | Pparg-F | TGTCGGTTTCAGAAGTGCCTTG | Pparg-R | TTCAGCTGGTCGATATCACTGGAG |
|  | Ap2-F | TGTGATGCCTTTGTGGGAACC | Ap2-R | CGTCCTGCGGTGATTTCATC |
|  | Cebpα-F | CAAGAACAGCAACGAGTACCG | Cebpα-R | GTCACTGGTCAACTCCAGCAC |
|  | Lpl-F | AGGTCATCTTCTGTGCTAGG | Lpl-R | ATGCTGGAAGACCTGCTATG |
|  | Perilipin-F | ATGTCTAGCAATGGTACAGATGC | Perilipin-R | CGTGGAACTGATAAGAGGCAGG |
|  | IL-2-F | TGGAGCAGCTGTTGATGGACCTAC | IL-2-R | AGATGATGCTTTGACAGAAGGCTATC |
|  | IL-4-F | GGGACGCCATGCACGGAGATG | IL-4-R | TGCGAAGCACCTTGGAAGCCC |
|  | Il-6-F | CCACTTCACAAGTCGGAGGCTTA | Il-6-R | CCAGTTTGGTAGCATCCATCATTTC |
|  | Il-10-F | GCATGGCCCAGAAATCAAGG | Il-10-R | GAGAAATCGATGACAGCGCC |
|  | Ifnγ-F | ATCTGGAGGAACTGGCAAAA | Ifnγ-R | TTCAAGACTTCAAAGAGTCTGAGGTA |
|  | Tnfα-F | TATGGCCCAGACCCTCACA | Tnfα-R | GGAGTAGACAAGGTACAACCCATC |
| Human | β-ACTIN-F | CCAGCACAATGAAGATCAAGAT | β-ACTIN-R | AGAAAGGGTGTAACGCAACTAA |
|  | LPL-F | TCATTCCCGGAGTAGCAGAGT | LPL-R | GGCCACAAGTTTTGGCACC |
|  | PPARg-F | TGTCGGTTTCAGAAGTGCCTTG | PPARg-R | TTCAGCTGGTCGATATCACTGGAG |
